# Hyperforin ameliorates neuroinflammation and white matter lesions by regulating microglial VEGFR_2_ /SRC pathway in vascular cognitive impairment mice

**DOI:** 10.1111/cns.14666

**Published:** 2024-03-11

**Authors:** Xin Gao, Jingjing Chen, Ge Yin, Yanqun Liu, Zhengsheng Gu, Rui Sun, Xu Sun, Xuehao Jiao, Ling Wang, Nuo Wang, Yanbo Zhang, Yuting Kan, Xiaoying Bi, Bingying Du

**Affiliations:** ^1^ Department of Neurology, Shanghai Changhai Hospital Second Military Medical University/Naval Medical University Shanghai China; ^2^ Department of Psychiatry, Faculty of Medicine and Dentistry University of Alberta Edmonton Alberta Canada

**Keywords:** hyperforin, microglia polarization, neuroinflammation, vascular cognitive impairment, white matter lesions

## Abstract

**Aim:**

To explore the neuroprotective potential of hyperforin and elucidate its underlying molecular mechanisms involved in its therapeutic effects against vascular cognitive impairment (VCI).

**Methods:**

The active compounds and possible targets of *Hypericum perforatum* L. that may be effective against VCI were found by network pharmacology in this research. We utilized bilateral common carotid artery occlusion (BCCAO) surgery to induce a VCI mouse model. Morris water maze (MWM) and Y‐maze tests were used to assess VCI mice's cognitive abilities following treatment with hyperforin. To evaluate white matter lesions (WMLs), we utilized Luxol fast blue (LFB) stain and immunofluorescence (IF). Neuroinflammation was assessed using IF, western blot (WB), and enzyme‐linked immunosorbent assay (ELISA). The effects of hyperforin on microglia were investigated by subjecting the BV2 microglial cell line to oxygen–glucose deprivation/reperfusion (OGD/R) stimulation. The expressions of VEGFR_2_, p‐SRC, SRC, VEGFA, and inflammatory markers including IL‐10, IL‐1β, TNF‐α, and IL‐6 were subsequently assessed.

**Results:**

The VEGFR_2_/SRC signaling pathway is essential for mediating the protective properties of hyperforin against VCI according to network pharmacology analysis. In vivo findings demonstrated that hyperforin effectively improved BCCAO‐induced cognitive impairment. Furthermore, staining results showed that hyperforin attenuated WMLs and reduced microglial activation in VCI mice. The hyperforin treatment group's ELISA results revealed a substantial decrease in IL‐1β, IL‐6, and TNF‐α levels. According to the results of in vitro experiments, hyperforin decreased the release of pro‐inflammatory mediators (TNF‐α, IL‐6, and IL‐1β) and blocked microglial M1‐polarization by modulating the VEGFR_2_/SRC signaling pathway.

**Conclusion:**

Hyperforin effectively modulated microglial M1 polarization and neuroinflammation by inhibiting the VEGFR_2_/SRC signaling pathways, thereby ameliorating WMLs and cognitive impairment in VCI mice.

## Graphical abstract

Our study systematically examined the underlying mechanisms of *Hypericum perforatum* L. in the vascular cognitive impairment (VCI) model using network pharmacology. Our experiments demonstrated that hyperforin, the most potent ingredient in *H. perforatum* L., ameliorates microglial activation, white matter lesions, and ultimately improves cognitive function in VCI mice through the VEGFR_2_/SRC pathway.

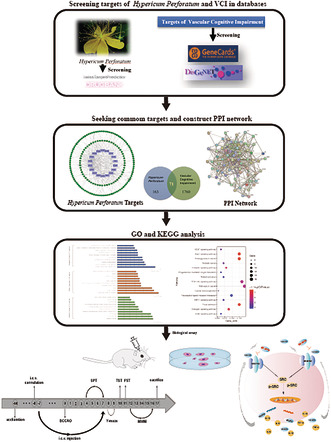

## 1 INTRODUCTION

Vascular cognitive impairment (VCI) is a major contributor to the prevalence of dementia, ranking as the second most prevalent cause and contributing to approximately 15%–30% of diagnosed cases. Starting with subjective cognitive decline and progressing to dementia, it encompasses the full range of vascular brain pathologies that affect cognition.^1^, ^2^ The etiology is multifactorial, including small vessel diseases and large vessel infarcts. However, the exact pathogenesis of VCI remains elusive. Neuroinflammation, chronic hypoperfusion, and hypoxia are all implicated in the pathogenesis of VCI.^3^ In the present time, the treatment of vascular diseases and management of risk factors are the primary approaches to managing VCI. However, there are limited pharmacological options available to ameliorate the disease. Therefore, novel treatment strategies for VCI are urgently needed.

The pathogenesis of VCI is a complex process, in which white matter lesions (WMLs) represent the most significant pathological changes. The “inflammatory mechanism” hypothesis, widely accepted as the dominant theory of VCI, has a crucial function in the processes of degradation and regeneration of myelin.^4^ Our prior clinical and animal studies on VCI have consistently shown the crucial involvement of microglial activation and concomitant neuroinflammation in the progression of WMLs and cognitive dysfunction.^5^, ^6^ Moreover, our study has shown that distinct inhibition of microglial activation and neuroinflammatory response in the hippocampus leads to differential improvement in myelin repair and ultimately enhances cognitive function in VCI mice.^7^ Neuroinflammation has the potential to cause WMLs, which are responsible for cognitive dysfunction.^4^, ^8^ Microglia, the resident macrophages in the central nervous system (CNS), play a pivotal role in regulating neuroinflammatory responses during the pathogenesis of VCI.^9^, ^10^ Microglia can exhibit two distinct phenotypes: the classical activation M1 phenotype, known for its pro‐inflammatory properties, and the alternative activation M2 phenotype, which is associated with anti‐inflammatory properties.^11^ Multiple studies have investigated the polarization of microglia, as it may contribute to the amelioration of neurodegenerative diseases by suppressing neuroinflammation.^12^, ^13^

Vascular endothelial growth factor A (VEGFA), often abbreviated as VEGF, is recognized as a key player in mediating physiological angiogenesis. However, VEGFA is also responsible for acute and chronic vascular hyperpermeability by phosphorylating VEGF receptor 2 (VEGFR_2_), thereby activating SRC and its downstream pathways.^14^ The secretion of VEGFA by neural progenitor cells can enhance the chemotaxis, proliferation, and phagocytosis of microglia. A study has conclusively demonstrated that the function of the SRC family (SFKs) is necessary and sufficient to trigger a pro‐inflammatory signature and phagocytosis in microglia.^15^ The actions of SFKs are crucial in regulating vital homeostatic functions of microglia, such as environmental sensing, phagocytosis, and the secretion of inflammatory mediators. However, microglia may become polarized into a deleterious phenotype when SRC is excessively activated.^16^

The medicinal plant *Hypericum perforatum* L. (as documented by World Flora Online), commonly known as Lianqiao in the Chinese Pharmacopeia, has a lengthy tradition of addressing depressive disorders^17^ and exhibits multiple pharmacological properties, some of which include antiviral, antitumor, anti‐inflammatory, and anti‐dementia properties.^18^, ^19^ During medical practice, the *Shugan Jieyu* capsule, composed of *H. perforatum* L. and *Ciwujia*, is widely employed for depression treatment. Contrary to what was previously thought to be the effect of hypericin, hyperforin, a major constituent found in *H. perforatum* L., has been recognized as a fundamental compound due to its antidepressant properties.^20^, ^21^ Previous research indicates that hyperforin can prevent spatial memory deficits in Alzheimer's disease (AD) by reducing astrogliosis and microglia activation.^22^, ^23^ Moreover, immune‐inflammatory responses of microglia, which contribute to the advancement of neuropathological conditions, are also impacted by hyperforin.^24^

The advent of bioinformatics, systems biology, and pharmacology has led to the emergence of network pharmacology as an invaluable framework for exploring the mechanisms and pharmacological properties of Traditional Chinese Medicine (TCM).^25^ Consequently, this study sought to systematically examine the underlying mechanisms of *H. perforatum* L. in the VCI model using network pharmacology. Our results demonstrated that hyperforin, the potent ingredient in *H. perforatum* L., ameliorates microglial activation, WMLs and ultimately improves cognitive function in VCI mice through the VEGFR_2_/SRC pathway.

## 2 MATERIALS AND METHODS

### 2.1 Network pharmacology analysis

#### 2.1.1 Active components and targets of *H. perforatum* L.

The main active components were screened by the Traditional Chinese Medicine Information Database (TCMID, https://www.bidd.group/TCMID/) and Traditional Chinese Medicine System Pharmacology Database (TCMSP, http://tcmspw.com/tcmsp.php) and literature review.^26^ Every compound referenced here has a canonical name that corresponds to its PubChem ID. The DrugBank database (https://www.drugbank.ca/)^27^ and the Swiss Target Prediction database (http://www.swisstargetprediction.ch/)^28^ were employed to identify the targets of the compounds.

#### 2.1.2 Disease targets

To pinpoint targets associated with VCI, the keywords “vascular cognitive impairment” or “vascular dementia” were searched through GeneCards (https://www.genecards.org)^29^ and DisGeNET (https://www.disgenet.org/home/)^30^ databases. The VCI target library was eventually created by merging the target data from the previously described databases and removing duplicates.

#### 2.1.3 Identification of core targets

To identify potential targets for *H. perforatum* L. therapy in VCI, a Venn diagram was built using the *H. perforatum* L. targets and VCI‐related targets with the aid of Venny 2.1.0 (https://bioinfogp.cnb.csic.es/tools/venny/). The resulting common targets between *H. perforatum* L. and VCI were considered possible therapeutic targets.

#### 2.1.4 Protein–protein interaction (PPI) networks constructing

The overlapping targets were entered for additional analysis into the STRING database (https://string‐db.org/, Version: 11.0). “Homo sapiens” was used as the species definition, and a PPI network was created using a 0.400 confidence level. Subsequently, the network's topological properties were assessed using Cytoscape 3.10.0 software (https://cytoscape.org/). The node degree values of the core targets were then used to rank them.^31^

#### 2.1.5 Gene Ontology and pathway enrichment analysis of core targets

To further explore the molecular mechanism of the core targets, DAVID (https://david.ncifcrf.gov/) was used to carry out Kyoto Encyclopedia of Genes and Genomes (KEGG) pathway enrichment analysis and Gene Ontology (GO) enrichment analysis. A significance cutoff of *p* < 0.05 was chosen as the criteria for analysis. The results of the top 15 enriched pathways and top 10 biological processes were selected and visualized using a bubble plot and bar charts.

### 2.2 In vivo experimental procedures

#### 2.2.1 Animals

For our in vivo experiments, we acquired adult male ICR mice, each weighing 25–30 g, from the Animal Laboratory Center of Naval Medical University. During the experiment, the mice were kept in a standard animal room with a temperature of 22 ± 2°C and a humidity of 55 ± 5%. All food and water were freely available to the mice and they were subjected to a 24‐h circadian rhythm. Before conducting the experiments, a 1‐week acclimation period was provided for the mice in the animal room. The procedures carried out in accordance with Changhai Hospital's authorized protocol (CHEC (A.E) 2023‐027) and the suggested guidelines specified in the National Institutes of Health's (USA) Guide for the Care and Use of Laboratory Animals were carefully followed during the animal studies.

#### 2.2.2 Experimental drugs

The hyperforin (Item No. 19572, purity ≥95%) and the VEGFR_2_ inhibitor SU 5416 (S8442, purity ≥98%) were purchased from Cayman Chemical (Ann Arbor, MI, USA) and Sigma‐Aldrich (St. Louis, MO, USA), correspondingly. DMSO was used to solubilize hyperforin and SU5416 to produce concentration levels of 10 and 20 mM, respectively. Throughout the experiments, the dilutions of both compounds were prepared in a manner that ensured the final DMSO concentration did not exceed 1%.

#### 2.2.3 Groups and drug treatments

Seven days postacclimation, an intracerebroventricular (i.c.v.) cannula (RWD Life Science Co., Ltd, Shenzhen, China) was inserted in the brain's left lateral ventricle (situated 3.1 mm deep, 0.6 mm posteriorly, and 1.1 mm laterally from the bregma). Subsequently, the mice were allocated randomly into the following groups: (1) sham group with normal saline (NS) administration (Sham + NS), (2) VCI group with NS administration (VCI + NS), and (3) VCI group with hyperforin (HP) administration (VCI + HP). Hyperforin was dissolved in saline. The concentration of hyperforin used in the animal experiments was primarily based on the studies published previously.^32^, ^33^ Hyperforin at concentrations of 0.5 μg/μL (1 μL, HP‐L), 1.0 μg/μL (1 μL, HP‐H), or saline (1 μL, NS) was slowly delivered intraventricularly into the left ventricle each day from day‐7 to day 7, consecutively.

#### 2.2.4 Transient bilateral common carotid artery occlusion

In this study, transient bilateral common carotid artery occlusion (BCCAO) surgery was employed to establish a model of VCI, as we previously reported.^7^ Prior investigations have demonstrated that mice subjected to BCCAO not only manifest symptoms of depression and cognitive impairment but also exhibit neuropathological alterations, including blood–brain barrier disruption, white matter damage, and glial activation.^34^, ^35^, ^36^ After the mice were given intraperitoneal anesthesia with 50 mg/kg of pentobarbital, a cervical midline incision was performed to carefully inspect and segregate the vagus nerves that were in close proximity to the common carotid arteries. Microvascular clips were used to clamp both common carotid arteries for a duration of 60 min. Following the restoration of cerebral blood flow, the wound was carefully sutured. To ensure a consistent body temperature in the mice, all surgical procedures were conducted on a thermal blanket. Except for carotid artery occlusion, the Sham + NS group experienced the same interventions.

#### 2.2.5 Animal behavioral tests

Due to the potentially weakened state of the mice after BCCAO surgery, we carefully planned the experimental order. First, we conducted the sucrose preference test and the Y‐maze test on the mice. Subsequently, after the neck wounds of the mice had healed and returned to their normal state, which approximately took about 10 days after the operation, we performed the tail suspension test, forced swimming test, and Morris water maze test. Furthermore, considering the potential influence of the Morris water maze on the forced swimming test, we performed the forced swimming test preceding the Morris water maze test. The experimental design flowchart is presented in Figure 2.

#### 2.2.6 Sucrose preference test

The sucrose preference test (SPT) was employed to assess anhedonia, which is a fundamental symptom of depression.^37^ Reduced sucrose intake is thought to be influenced by anhedonia and diminished sensitivity to rewards. During the experiment, every mouse was kept in an individual cage, isolated from others. Two stages were involved in the test: (1) adaptation stage: each mouse was provided with two bottles of the same pure water on day 4, then one bottle each of clean water and 1% sucrose water on day 5, and the bottles' positions were changed 12 h later; (2) test stage: mice underwent a 24‐h deprivation of food and water following the adaptation phase. On day 7, two aforementioned bottles were again given to the mice. Sucrose water and pure water consumption were measured at 1, 6, and 24 h intervals, respectively, to calculate the sucrose preference index.

#### 2.2.7 Tail suspension test

The tail suspension test (TST) is commonly employed to evaluate the antidepressant efficacy of drugs. It was conducted on day 10 according to the protocols.^38^ Briefly, after 1 h of acclimatization, each mouse was suspended with their tails positioned 50 cm above the ground on a shelf. The immobility, representing “behavioral despair” was defied as mice gave up struggling the body and head in a relaxed state. The behaviors were recorded for 6 min by a camera (Shanghai Xinruan, China). In the final 4 min, two treatment‐blinded researchers measured the immobility.^39^

#### 2.2.8 Forced swimming test

The forced swimming test (FST) was established to measure depressive‐like behavior.^40^ Briefly, after 1 h of acclimatization, each mouse was placed into a clear cylinder that measured 12 cm in diameter, 23–25°C in temperature, and 16 cm in depth of water. Immobility is defined as a lack of movement, excluding those essential for maintaining their head above water level.^41^ The behaviors were recorded for 6 min by a camera (Shanghai Xinruan, China). In the final 4 min, two treatment‐blinded researchers measured immobility. Before returning to their cages, mice were carefully dried. To prevent any potential influence om mice, the water was changed between subjects.

#### 2.2.9 Y‐maze

Y‐maze is a popular method for evaluating cognitive skills, which is particularly useful for testing spatial position and orientation.^42^ The experiment was conducted on days 8 and 9 (Figure 2). There are three symmetrical arms on the device (5 cm wide, 10 cm high, and 35 cm long) in a Y‐shaped configuration, labeled A, B, and C, forming an equilateral triangle. Before the experiment, all mice underwent a 1‐h acclimation period in the experimental environment. During the spontaneous alternation experiment, the mice were gently introduced into one arm of the maze and given unrestricted exploration for 10 min. It was defined as a correct visit when the mice sequentially explored all three arms (e.g., A–B–C) without revisiting any individual arm more than once within three consecutive alternations. The rate of spontaneous alternation is calculated as [(Number of alternations)/(Total number of arm entries − 2)]. During the experiment of exploring the new arm, one of the arms was randomly closed off with clapboard and designated as the new arm from the others. The mice were introduced into the maze, beginning from the starting arm's starting point, and given 10 min to explore the two arms. After a 2‐h interval, the clapboard was opened to reveal the new arm, and the observation time was 5 min. The novelty index was calculated as the ratio of the total time spent investigating all three arms to the time spent exploring new arms. To minimize potential bias, the device was sanitized using 75% ethanol before each subsequent trial.

#### 2.2.10 Morris water maze

One behavioral test that is commonly used to study and assess memory and spatial learning is the MWM. The device comprises a round pool that is evenly partitioned into four quadrants, accompanied by a platform positioned approximately 1 cm beneath the water level. At the midpoint of the edge of the designated quadrant, the mice were introduced into the water during the hidden platform experiment. Each observation was conducted for 60 s. If the mice successfully found the platform and stayed for 5 s on it, the learning phase was considered complete, and latencies are the times taken to locate the platform. The maximum latency was 60 s if the mice could not find the platform in that time. The mice were then left for 10 s on the platform. The platform was removed after the animals had been trained for 4 days, and the mice were then allowed to locate the original platform. The amount of time they spent in the target quadrant and the number of times they traversed the platform were monitored within a 60‐s timeframe to determine their memory and learning skills. Data analysis was conducted using Shanghai Xinruan software (Shanghai, China).

#### 2.2.11 Collection of samples

After conducting the behavioral experiments, the mice were subjected to anesthesia using pentobarbital and then intracardially perfused with saline. After decapitation, the skull was dissected to expose the brain. The hippocampus tissue was then collected and placed in an Ep tube, storing it in a −80°C freezer for enzyme‐linked immunosorbent assay (ELISA) and western blot (WB). Other brains were fixed overnight using 4% paraformaldehyde, which was then embedded in paraffin and sectioned for subsequent staining analysis.

#### 2.2.12 Immunofluorescence staining

The immunofluorescence (IF) staining procedure was performed on the paraffin slices. The paraffin portions were first dewaxed and citrate‐repaired, then the brain slices were blocked for an hour at room temperature (RT) using a PBS solution containing 0.3% Triton X‐100 and 5% BSA. Subsequently, sections were allowed to incubate overnight with primary antibodies (MBP (1:1000, ABclonal, A11162); Neun (1:500, Abcam, Ab177487); Iba‐1 (1:500, Wako, 019‐19741)) at 4°C. After PBS washing, the secondary antibody (1:500, Invitrogen, A‐110081, A‐110121) was added for 1 h. Then the tissue sections were rinsed for 5 min with DAPI solution. As a final step, the sections were covered with coverslips by utilizing an antifade mounting medium to minimize fluorescence quenching. The obtained images were analyzed using Image‐Pro Plus software to quantify the number of microglia and neurons as well as the IntDen of the MBP staining after imaging by a fluorescent microscope (Olympus Corporation, Japan).

#### 2.2.13 Luxol fast blue stain

Pathological lesions in myelinated axons were identified using Luxol fast blue (LFB) staining.^43^ In brief, the sections were deparaffinized and then subjected to incubation in an LFB solution at 60°C for 1 h. After the termination of staining, the slides were sealed with neutral resin. Then the integrated optical density (IOD) was acquired by Image‐Pro Plus (Media Cybernetics, USA).

### 2.3 In vitro experimental procedures

#### 2.3.1 Drug treatment, oxygen–glucose‐deprivation/reperfusion, and cell culture

Studies have demonstrated that BV2 cell line exhibits a phenotype akin to primary brain microglia, expressing nonspecific esterase activity, phagocytic ability, and lacking peroxidase activity.^44^ For our in vitro experiments, we seeded BV2 microglial cells, which were acquired from Qui Cell firm located in Shanghai, China, onto a six‐well plate (1 × 10^6^ cells/well). Thereafter, 10% fetal bovine serum and 1% Penicillin–Streptomycin (PS) were added to a DMEM medium to culture BV2 cells. Oxygen–glucose deprivation/reperfusion (OGD/R) was employed to simulate acute cerebral ischemia‐hypoxia reperfusion and induce microglial activation.^45^, ^46^ In a specialized hypoxic incubator, the cells are subjected to a glucose‐free medium for 2 h to induce OGD/R. The hypoxic incubator was filled with a gas mixture with 94% N_2_, 5% CO_2_, and 1% O_2_. Subsequently, the cells were returned to standard culture conditions and incubated in normal culture media for 24 h. During and after the OGD/R treatment, the cells were subjected to incubation with or without 0.5 and 1.0 μM hyperforin or 5 and 10 μM SU5416. The concentration selections were based on previous literatures, with some modifications.^47^, ^48^ After the OGD/R experiment, the cell supernatant and protein were collected for corresponding experiments.

#### 2.3.2 Cell viability assay

With the use of the Cell Counting Kit‐8 (CCK‐8) (Yeasen Biotechnology Co., Ltd. Shanghai, China), the impact of the hyperforin on BV2 cells was evaluated. After plating the BV2 cells (5 × 10^3^ cells/well), they were allowed to incubate for 24 h. The cells were then cultured for a further 24 h at different doses of hyperforin (0, 0.25, 0.5, 0.75, 1.0, and 2.0 μM).^49^ Then, 10 μL CCK‐8 solution was added to all the wells carefully and incubated for 2 h. The absorbance values at 450 nm were recorded.

#### 2.3.3 ELISA

As instructed by the manufacturer, ELISA Kits (Shanghai Enzyme‐linked Biotechnology, Shanghai, China) were applied to measure the levels of IL‐10, TNF‐α, IL‐6, and IL‐1β in the supernatants of BV2 cell culture and the hippocampus tissue. Briefly, the appropriate amount of sample was added, and then the reaction solution was added in sequence. At last, the absorbance values at 450 nm were recorded.

#### 2.3.4 Western blot analysis

Protease and phosphatase inhibitor‐containing RIPA buffer was used to lyse BV2 cells and hippocampal tissues to measure protein expression. Subsequently, 20 μL of total cell lysates or tissue lysates were loaded onto 4%–12% precast polyacrylamide gel electrophoresis (PAGE) gels to separate proteins based on size. Following their transfer to a PVDF membrane (Millipore, CA), the separated proteins were blocked using skim milk for 90 min at room temperature. The membranes were thereafter incubated overnight at 4°C with primary antibodies against MBP (1:1000, ABclonal, A11162), p‐SRC(1:1000, Cell Signaling Technology, D7F2Q), SRC (1:1000, Cell Signaling Technology, 36D10), VEGFA (1:1000, ABclonal, A17877), VEGFR_2_ (1:200, Santa Cruz, sc‐22165), CD86(1:1000, ABclonal, A19026), ARG1 (1:1000, ABclonal, A4923), iNOS (1:1000, ABclonal, A3774), and β‐actin(1:1000, ABclonal, AC026), respectively. The secondary antibody was introduced to incubate on the membranes for 1 h at RT following three TBST washes. The signals were visualized by ImageQuant LAS 4000 (Cytiva, Japan) and measured by ImageJ.

### 2.4 Statistical analysis

The data were displayed as the mean ± standard error of the mean (SEM). For graphing and data analysis, SPSS 26 (SPSS, Chicago, Illinois, USA) and GraphPad Prism 9 (GraphPad Software Inc, San Diego, CA, USA) were performed. The normality of data was assessed using the Shapiro–Wilk normality test. To assess the significant differences between the groups, data that exhibited a normal distribution were analyzed using a one‐way ANOVA with post hoc LSD‐t test. Alternatively, the Kruskal–Wallis test was employed to analyze the group differences when the distribution was not parametric. The escape delay in MWM was investigated using repeated measures ANOVA. *p* less than 0.05 was considered significant.

## 3 RESULTS

### 3.1 Core components and potential targets of *H. perforatum* L. regulating VCI

**FIGURE 1:**
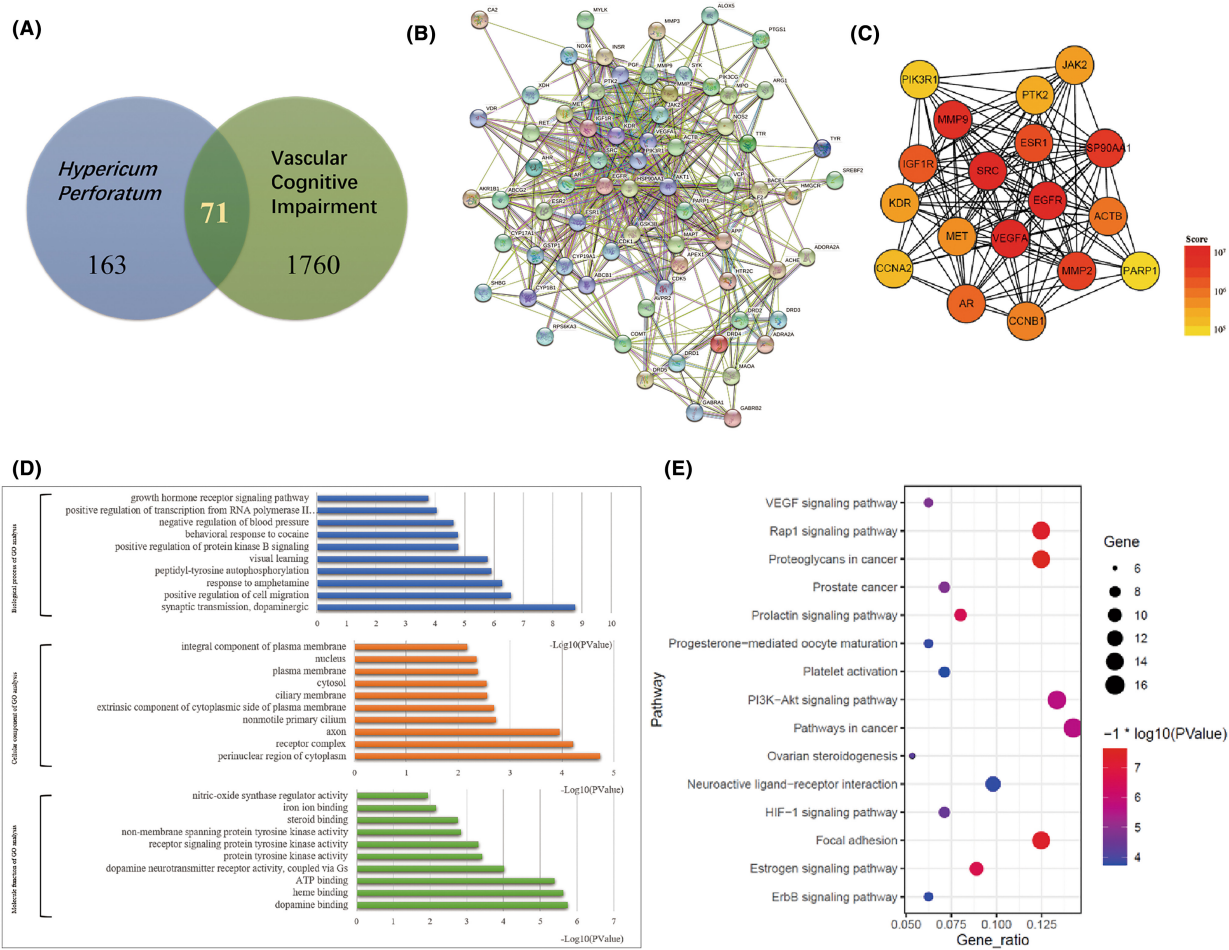
Protein–protein interaction (PPI) network, Gene Ontology (GO) enrichment, and Kyoto encyclopedia of genes and genomes (KEGG) pathway analyses of potential targets. (A) Venn diagram illustrating the pathogenesis‐related targets of VCI and *Hypericum perforatum* L. (B) PPI network of 71 potential target genes. (C) The highest score targets according to the correlation ranking results identified by CytoHubba. (D) The top 10 biological processes associated with GO enrichment analysis's MF, CC, and BP. (E) Bubble diagram of top15 KEGG enrichment pathways.

After removing duplicate entries, a total of 18 active constituents of *H. perforatum* L. were discovered (Table S1), and hyperforin was one of the main constituents. Meanwhile, 163 potential targets of *H. perforatum* L. and 1760 acknowledged VCI‐associated genes were eventually identified from the database. The Venn diagram revealed 71 potential target genes in total (Figure 1A). Subsequently, a PPI network was formulated utilizing the STRING database and it contained 496 edges and 71 nodes. The node degree average was 14 and the local clustering coefficient average was 0.563 (Figure 1B). CytoHubba's correlation ranking revealed that the highest score targets were SRC, VEGF, and EGFR (Figure 1C). Subsequently, all the potential targets were analyzed by DAVID database and annotated based on biological processes (BP), cellular components (CC), and molecular functions (MF) (Figure 1D). Pathway study using KEGG further revealed that VEGF signaling pathway could potentially have a crucial role in the treatment of VCI (Figure 1E). In light of the above results and existing literature, we developed the hypothesis that hyperforin, the active compound of *H. perforatum* L. could improve the cognitive abilities of VCI through regulating the VEGFR_2_/SRC signaling pathway.

### 3.2 Hyperforin effectively alleviates the cognitive impairment in VCI mice

**FIGURE 2:**
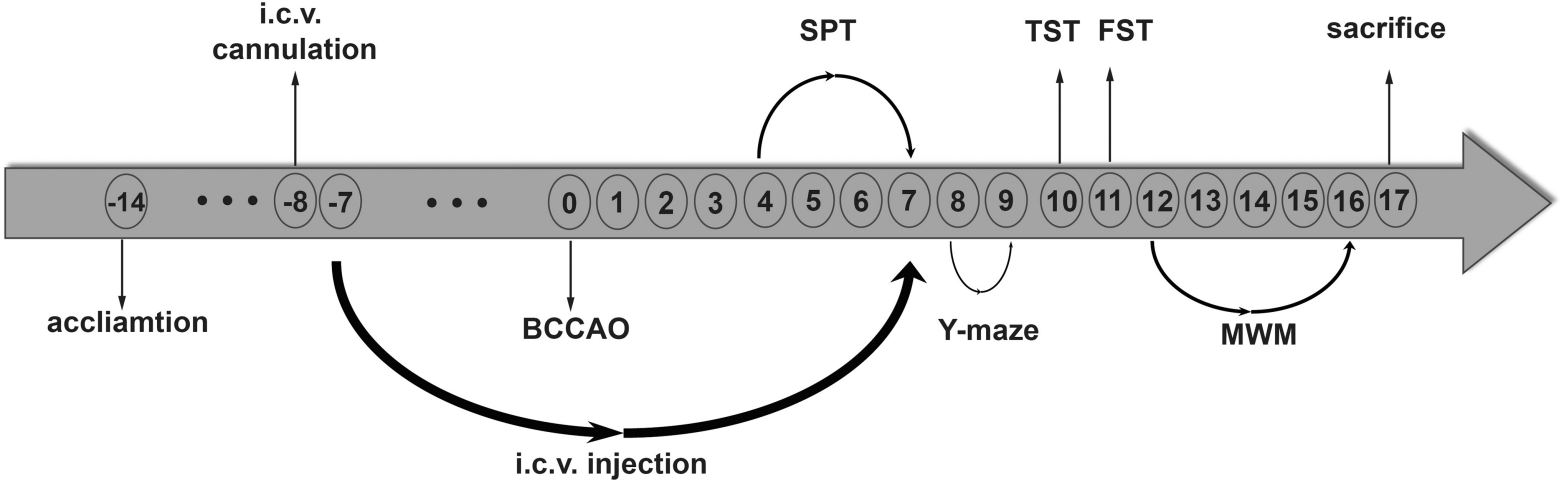
Flowchart of the experiment. BCCAO, bilateral common carotid artery occlusion; FST, forced swimming test; i.c.v., intracerebroventricular; MWM, Morris water maze; SPT, sucrose preference test; TST, tail suspension test.

Figure 2 depicts the timeline of this experiment. To investigate the optimal dosage of hyperforin for treating cognitive deficits in BCCAO‐induced VCI mice, we designed two doses of 0.5 μg/μL (HP‐L) and 1.0 μg/μL (HP‐H) and assessed their cognitive abilities using the Y‐maze and MWM. We observed a significantly higher mortality rate in the higher concentration group (30%) compared to the lower concentration group (10%) during the BCCAO procedure. The results showed that both concentrations of hyperforin improved cognition in VCI mice (Figure S1A,B,D,F). Additionally, a comparative analysis of swimming speeds among the four groups of mice revealed no differences in motor function (Figure S1C). However, the higher concentration group demonstrated less improvement in terms of platform crossing times in the MWM test compared with the lower concentration group (Figure S1F). Additionally, western blot analysis showed that the VCI + HP‐L group's hippocampus MBP expression was greater than that of the VCI + HP‐H group (Figure S1G,H). These findings suggest that the lower concentration hyperforin exhibited more significant cognitive improvement with less white matter damage. Therefore, we selected the lower concentration dosage for subsequent experiments.

**FIGURE 3:**
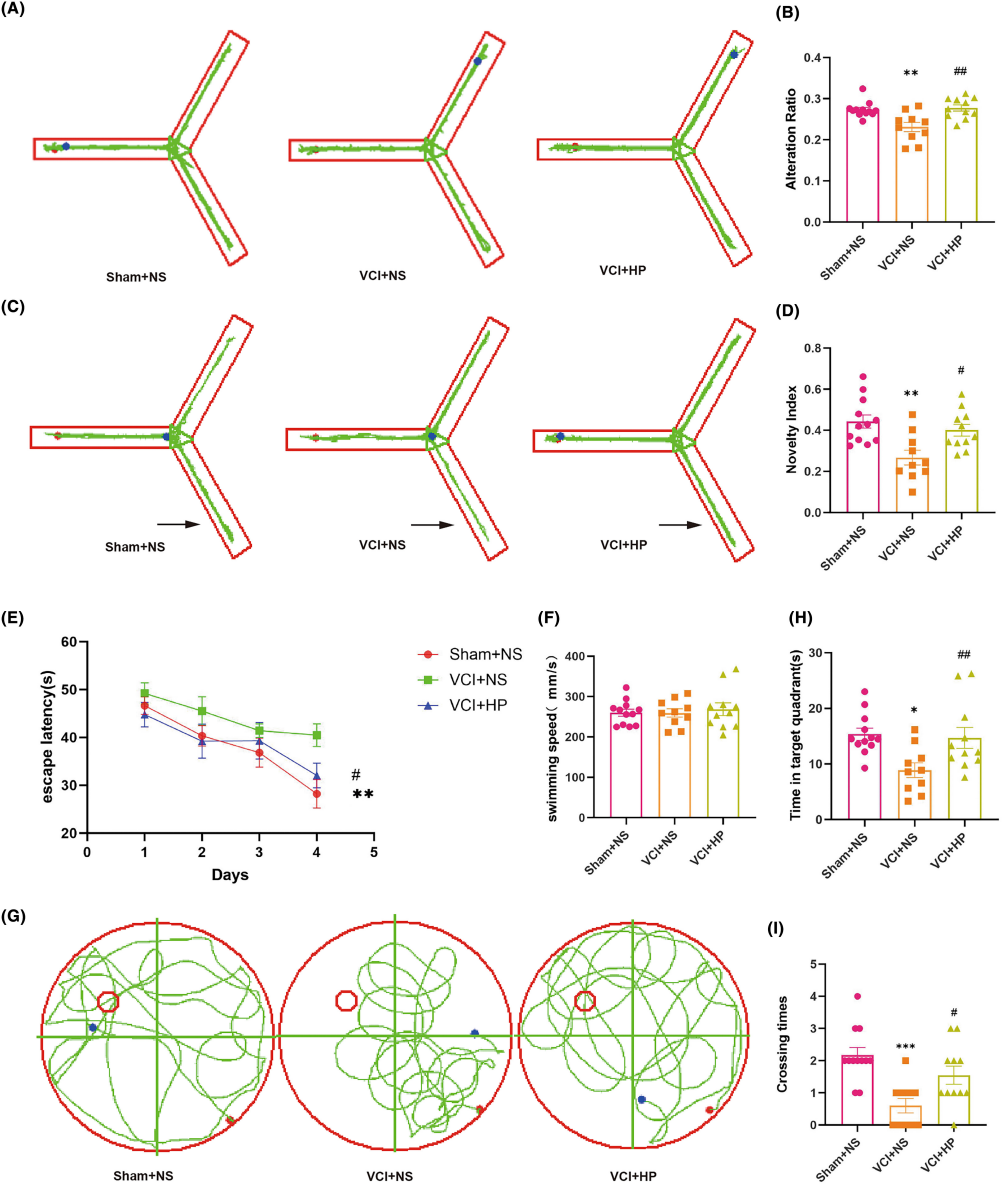
Hyperforin ameliorates cognitive impairment in vascular cognitive impairment (VCI) mice as demonstrated by the Y‐maze and Morris water maze (MWM) experiment. (A) The track plot of the spontaneous alternation experiment in the Y‐maze test. (B) The Alternations ratio in Y‐maze test. (C) The track plot of the new arm exploration experiment in the Y‐maze test. (D) The novelty index in Y‐maze test. (E) The escape latency period of the MWM test. (F) The swimming speeds of the mice. (G) The duration of stay in the MWM target quadrant on day 5. (H) The track plot of the MWM test on day 5. (I) The crossing times of the platform in the MWM test. The data are depicted as mean ± SEM (*n* = 10–12). *p < 0.05, **p < 0.01, and ***p < 0.001 versus Sham + NS group; ^#^ *p* < 0.05, ^##^ *p* < 0.01 versus VCI + NS group.

As illustrated in Figure 3A,B, compared to the Sham + NS group, the VCI + NS group exhibited a noteworthy decline in cognitive function and a significant reduction in the alternation ratio (*p* < 0.01). Additionally, the working memory ability was remarkably improved when VCI mice were treated with hyperforin (*p* < 0.01). The novelty index of the VCI + NS group demonstrated a significant decrease (*p* < 0.01) in comparison to the Sham + NS group. Similarly, treatment with hyperforin demonstrated a significant restoration of spatial reference memory functions (*p* < 0.05, Figure 3C,D). The MWM experiment is used to assess mice's spatial memory and learning. Specifically, the mice in the VCI + NS group exhibited a markedly higher escape latency (*p* < 0.01) compared with the Sham + NS group. This may indicate a decline in the VCI mice's capacity to learn and remember. Conversely, the VCI + HP mice showed a reduced escape latency following HP administration (*p* < 0.05) (Figure 3E). Additionally, swimming speeds were compared among all groups, and the results indicated no disparity in motor function (Figure 3F, *p* = 0.365). Figure 3G–I presented the results of the space exploration experiment. Compared to the Sham + NS group, the VCI + NS mice displayed a decrease in time spent in the target quadrant (*p* < 0.05) and a drop in platform crossings (*p* < 0.001). The hyperforin‐treated mice showed a significant increase in the frequency of platform crossings (*p* < 0.05) and spent more time in the target quadrant (*p* < 0.05) compared to the VCI + NS animals. These findings clearly suggested that hyperforin treatment has the potential to ameliorate cognitive impairments in VCI mice.

### 3.3 Hyperforin moderately alleviates the depressive behaviors in VCI mice

The assessment of depression‐like behaviors was conducted using the TST, FST, and SPT. The VCI + NS group showed a significantly increased immobility time in TST, along with a reduced 24‐h sucrose consumption during the SPT compared with the Sham + NS group (both *p*s < 0.05) (Figure S2A,E). However, VCI mice administrated with hyperforin showed a remarkable shorter immobility time than VCI + NS group in TST (*p* < 0.05) (Figure S2A). Additionally, mice in the VCI + HP group exhibited a significant rise in 24‐h sucrose consumption in contrast to those in the VCI + NS group (*p* < 0.05, Figure S2E). On the contrary, there were no differences between the groups in FST, 1‐h sucrose consumption, and 6‐h sucrose consumption (Figure S1B–D). Overall, these findings suggest that VCI mice exhibited anhedonia and depressive‐like behaviors, while treatment with hyperforin could moderately alleviate the depressive behaviors.

### 3.4 Hyperforin significantly reversed the white matter lesions in VCI mice

**FIGURE 4:**
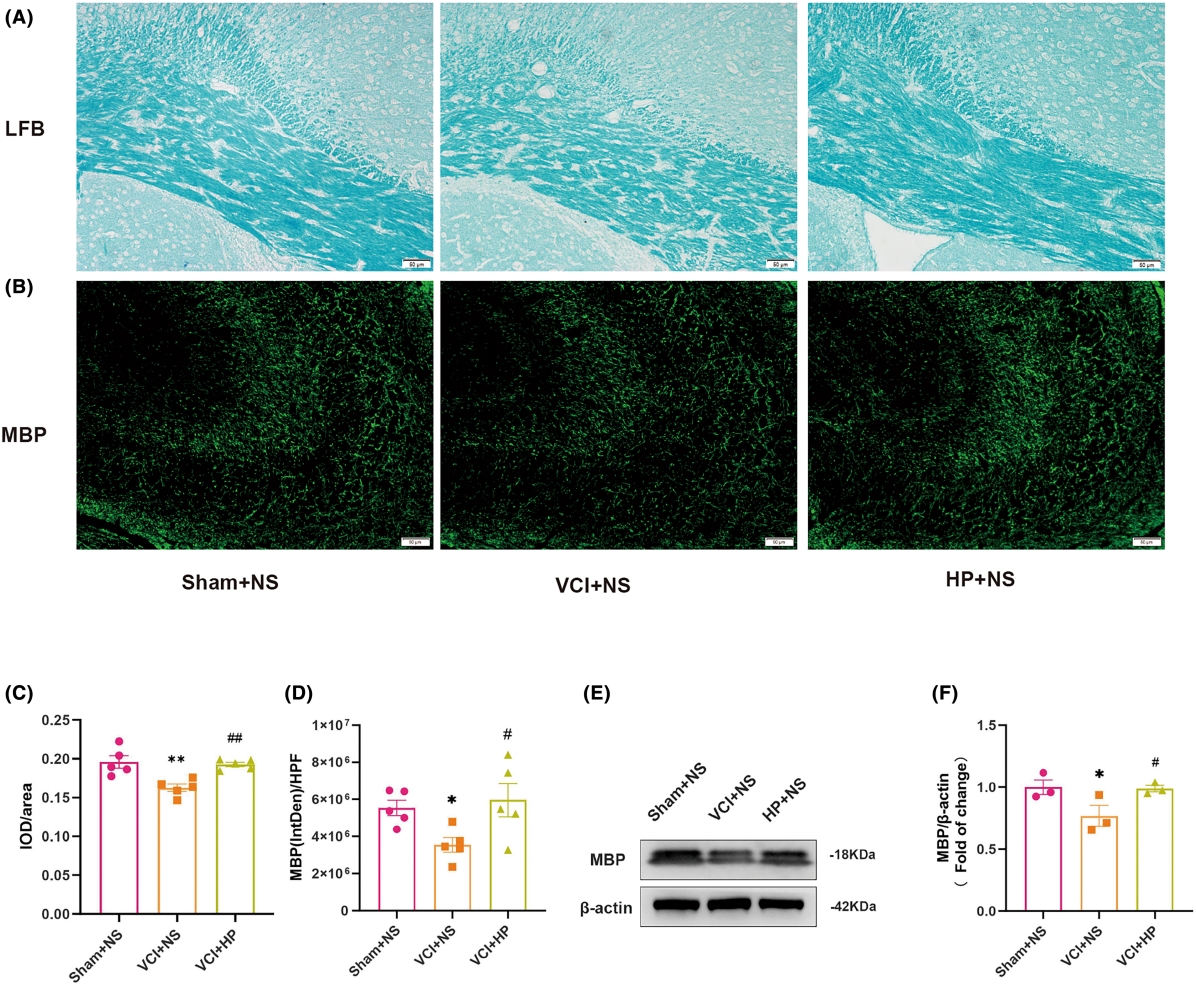
Hyperforin reversed the white matter lesions in vascular cognitive impairment (VCI) mice. (A, B) Images of MBP immunofluorescence in the CA3 region of the hippocampus and Luxol fast blue (LFB) staining in the corpus callosum. (C, D) Analysis of myelination quantitatively measured by IOD of LFB in the corpus callosum and IntDen of MBP immunofluorescence in hippocampal CA3 area. (E, F) Immunoreactive bands and quantitative analysis of WB of MBP and β‐Actin. Data are depicted as mean ± SEM (*n* = 5 or 3). *p < 0.05, **p < 0.01 versus Sham + NS group; ^#^ *p* < 0.05, ^##^ *p* < 0.05 versus VCI + NS group.

To assess the extent of WMLs, the myelin sheaths in the hippocampus and corpus callosum were analyzed using MBP and LFB staining, respectively. The LFB staining results demonstrated noticeable demyelination in the corpus callosum of VCI + NS group (*p* < 0.01), whereas hyperforin treatment could significantly protect against WMLs in VCI mice (*p* < 0.01) (Figure 4A,C). Similarly, as depicted in Figure 4B,D, MBP staining showed remarkable WMLs in the hippocampus of the VCI + NS mice relative to the Sham + NS mice (*p* < 0.05). Importantly, the administration of hyperforin significantly ameliorated demyelination in the hippocampus (*p* < 0.05) (Figure 4B,D). Furthermore, WB analysis indicated a reduction in MBP expression in the VCI + NS mice when contrasted with the Sham + NS mice (*p* < 0.05), while hyperforin treatment induced an elevation of MBP expression (*p* < 0.05, Figure 4E,F).

Additionally, Neun IF staining was employed to quantify neurons in the hippocampus. In contrast to Sham + NS mice, the VCI + NS group was found to have a potential reduction in neurons in the hippocampal CA3 region. However, it is noteworthy that no significant difference was found at a statistical level (*p* = 0.4151, Figure S3). Meanwhile, there was no significant difference in the number of Neun^+^ cells between the VCI + NS and VCI + HP groups (*p* = 0.4628, Figure S3).

### 3.5 Hyperforin remarkably inhibited microglial activation and neuroinflammation in VCI mice

**FIGURE 5:**
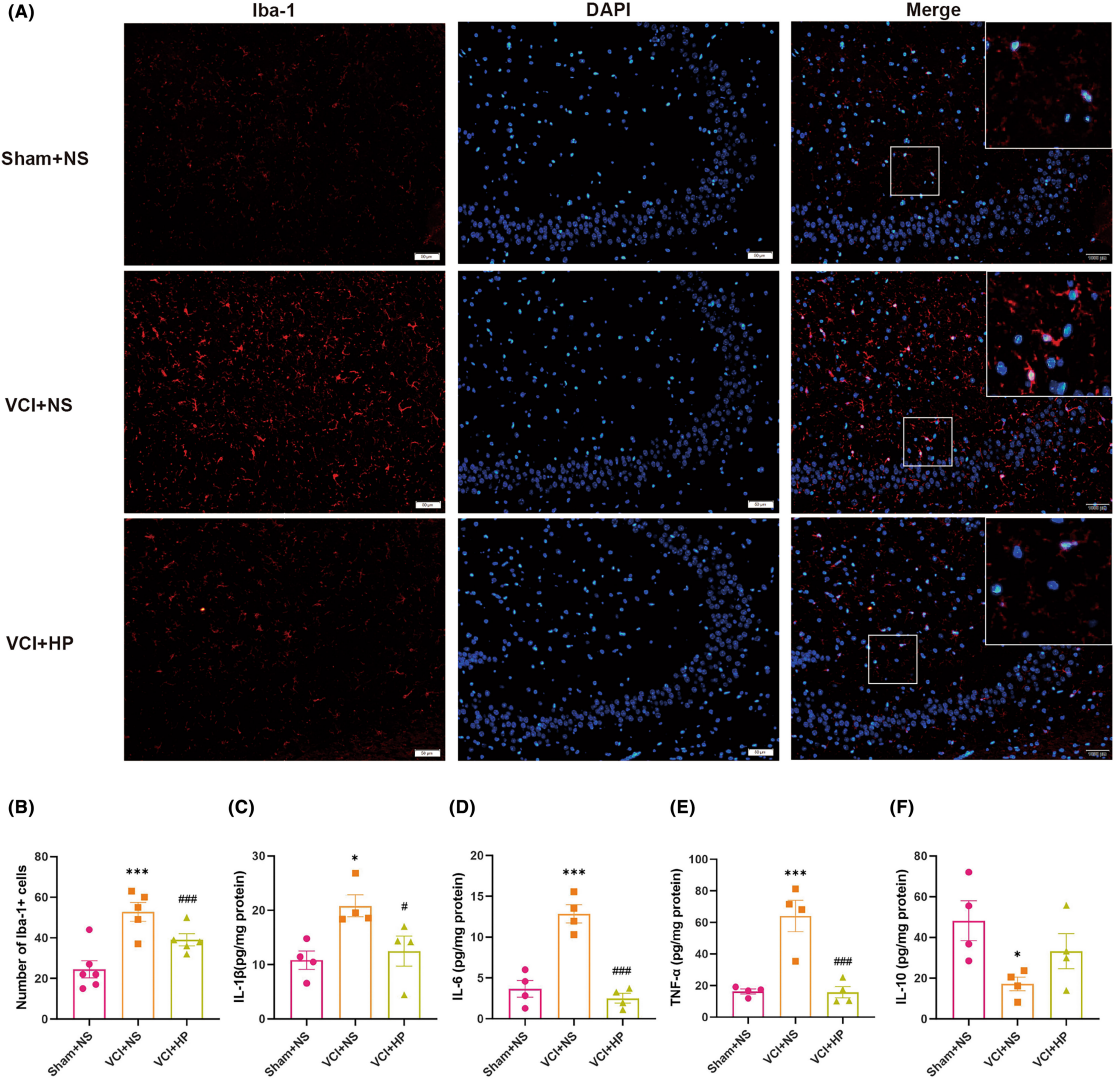
Hyperforin inhibited microglial activation and neuroinflammation in vascular cognitive impairment (VCI) mice. (A) Iba‐1^+^ immunofluorescence in the hippocampus CA3 region as seen in representative images. (B) Quantification analysis of Iba‐1^+^ cells in hippocampal CA3 area. (C–F) ELISA analysis of hippocampal contents of IL‐1β, IL‐6, TNF‐α, and IL‐10. Mean ± SEM is used to express data (*n* = 5–6 or *n* = 5, respectively). *p < 0.05, and ***p < 0.001 versus Sham + NS group; ^#^ *p* < 0.05, and ^###^ *p* < 0.001 versus VCI + NS group.

After day 17, we assessed how hyperforin affected neuroinflammation and microglia activation (Figure 5). Iba‐1 antibody was adopted to label microglia in the hippocampus. In contrast to the Sham + NS group, we discovered that the VCI + NS group had a notably higher quantity of Iba‐1^+^ cells, with a *p* value of less than 0.001. Nonetheless, the quantity of Iba‐1^+^ cells in VCI mice was dramatically decreased by the administration of hyperforin (*p* < 0.05) (Figures 5A and 4B).

To evaluate the inflammatory status in VCI mice, ELISA was employed to quantify inflammatory cytokine expression in the hippocampal tissue. The findings showed that IL‐1β, TNF‐α, and IL‐6 levels were considerably higher in VCI + NS group mice than in Sham + NS mice (all *p*s <0.05), whereas IL‐10 levels were markedly lowered (*p* < 0.05). Curiously, the VCI + HP group had a significant decrease in IL‐1β, TNF‐α, and IL‐6, in contrast to the VCI + NS group (Figure 5C–E). Likewise, treatment with hyperforin increased the concentrations of IL‐10, although there was no statistical difference (*p* = 0.176, Figure 5F).

### 3.6 SU5416 effectively suppressed microglial M1‐polarization by inhibiting VEGFR_2_ /SRC pathway in OGD/R‐induced BV2 cells

**FIGURE 6:**
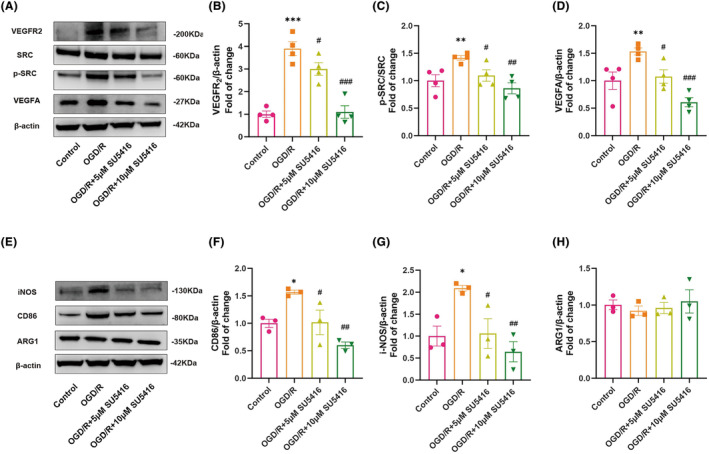
SU5416 suppressed microglial M1 polarization by inhibiting VEGFR_2_/SRC pathway in OGD/R‐induced BV2 cells. (A–D) Immunoreactive bands and quantitative analysis of western blot of VRGFR_2_, p‐SRC, SRC, VEGFA, and β‐Actin. (E–H) Immunoreactive bands and quantitative analysis of western blot of iNOS, ARG1, and β‐Actin. Mean ± SEM is used to express data (*n* = 4–5). *p < 0.05, **p < 0.01, and ***p < 0.001 versus control group; ^#^ *p* < 0.05, ^##^ *p* < 0.01, and ^###^ *p* < 0.001 versus OGD/R group.

To investigate the involvement of the VEGFR_2_/SRC pathway in microglia, BV2 cells receiving OGD/R were treated with SU5416, a VEGFR_2_ inhibitor. As shown in Figure 6A–C,E–G, OGD/R‐induced BV2 cells exhibited significantly increased expressions of VEGFR_2_, p‐SRC/SRC, CD86, and iNOS in contrast to the control group (all *p*s < 0.05). This suggests that the VEGFR_2_/SRC signaling pathway activation enhances microglial M1 polarization following OGD/R. Interestingly, when OGD/R microglial cells were further treated with SU5416, the levels of VEGFR_2_, p‐SRC/SRC, CD86, and iNOS declined dose‐dependently (all *p*s < 0.05). Additionally, VEGFA expression followed the same trend as other protein levels mentioned above (all *p*s < 0.05) (Figure 6D), indicating that microglial activation upregulated VEGFA concentration in BV2 cells. However, no significant differences in ARG1, a marker of microglial M2 polarization, were observed between the groups at a statistical level (*p* = 0.832) (Figure 6H).

### 3.7 Hyperforin significantly attenuated microglial M1 polarization by regulating VEGFR_2_ /SRC pathway in OGD/R‐induced BV2 cells

The CCK‐8 test was implemented to evaluate the viability of BV2 cells. To study the effects of hyperforin, BV2 cells were treated with different dosages (0, 0.25, 0.5, 0.75, 1.0, and 2.0 μM) at different time points. The results indicated that low‐dose hyperforin (0–0.75 nM) did not affect the activity of BV2 microglia cells following the 6, 12, 24, and 48‐h culture period (Figure S4). Conversely, there was a dosage‐ and time‐dependent reduction in cell viability when the hyperforin dosage was increased to 2.0 nM (dose of 2.0 nM: *p* = 0.287, *p* < 0.001, *p* < 0.001, *p* < 0.001 vs. vehicle group following 6, 12, 24, and 48‐h culture period; Figure S4). Therefore, doses of 0.5 and 1.0 nM were selected for subsequent OGD/R experiments.

**FIGURE 7:**
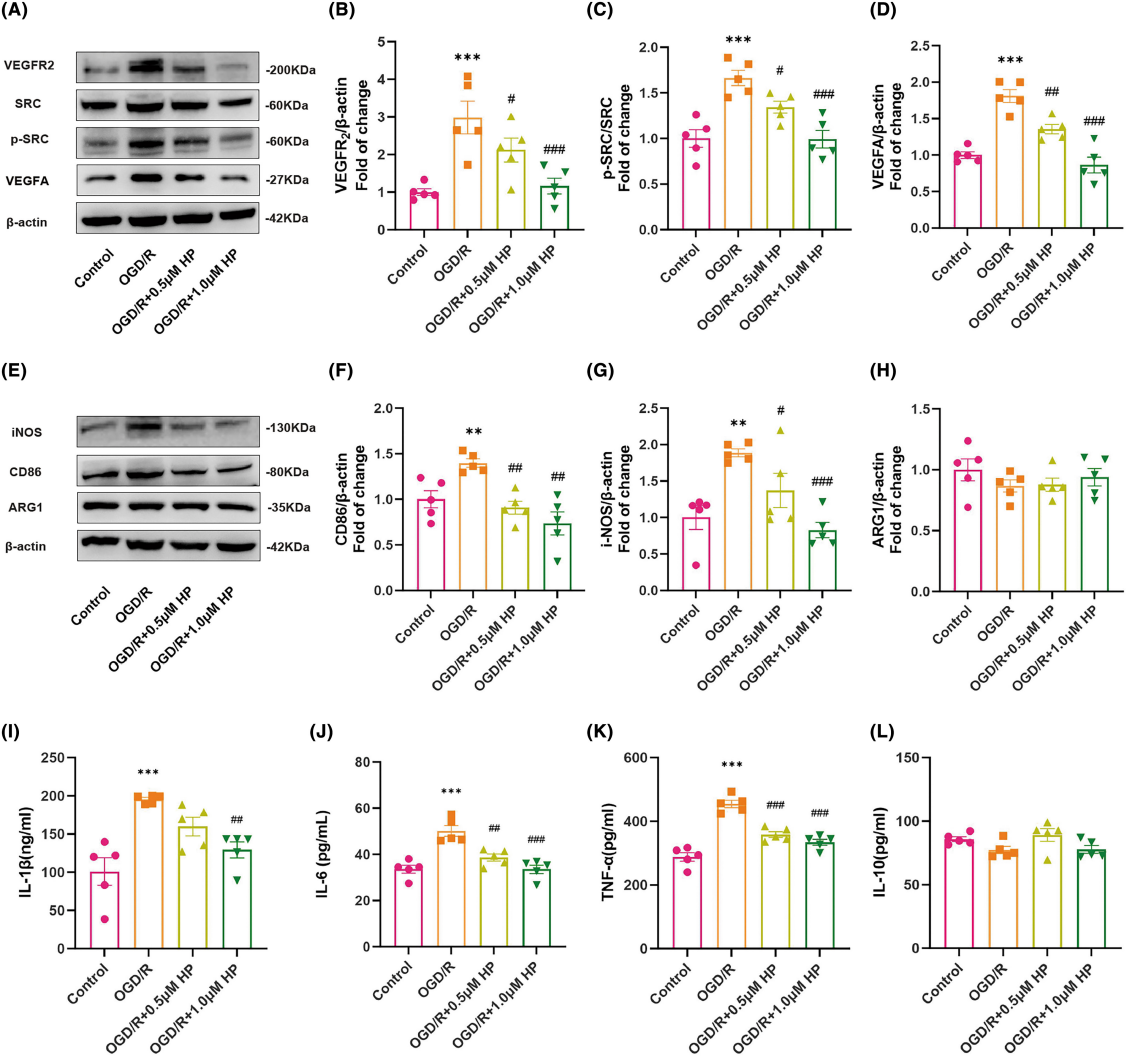
Hyperforin attenuated microglial M1 polarization by regulating VEGFR_2_/SRC pathway in OGD/R‐induced BV2 cells. (A–D) Immunoreactive bands and quantitative analysis of western blot of VRGFR_2_, p‐SRC, SRC, VEGFA, and β‐Actin. (E–H) Immunoreactive bands and quantitative analysis of western blot of CD86, iNOS, ARG1, and β‐Actin. (I–L) Expression levels of IL‐10, TNF‐α, IL‐6, and IL‐1β in the supernatants of BV2 cells. Mean ± SEM is used to represent data (*n* = 4–5). **p < 0.01, and ***p < 0.001 versus control group; ^#^ *p* < 0.05, ^##^ *p* < 0.01, and ^###^ *p* < 0.001 versus OGD/R group.

Subsequently, the impact of hyperforin on the VEGFR_2_/SRC pathway was examined through western blot analysis. The results indicated the increased expression of VEGFR_2_, p‐SRC/SRC, and VEGFA in BV2 cells receiving OGD/R (all *p*s < 0.001, Figure 7A–D). However, the administration of 0.5 and 1.0 μM hyperforin resulted in a dose‐dependent decrease in their expression (all *p*s < 0.05, Figure 7A–D). Additionally, the polarization state of BV2 cells was further investigated. The results showed that the OGD/R group had significant upregulation of CD68 and iNOS, markers of microglial M1‐polarization, in contrast to the control group (both *p*s < 0.01, Figure 7E–G). However, levels of these proteins were significantly downregulated after incubation with 0.5 and 1.0 μM hyperforin (all *p*s < 0.05, Figure 7E–G). Similarly, the expression of ARG1 between different groups was not statistically different (*p* = 0.620, Figure 7H). Furthermore, the results revealed a consistent elevation in the concentrations of IL‐1β, TNF‐α, and IL‐6 in the OGD/R group (all *p*s < 0.001), indicating microglia‐associated neuroinflammation. Conversely, treatment with hyperforin dose‐dependently reversed this neuroinflammatory response (all *p* < 0.05 for 1.0 μM hyperforin, Figure 7I–K). Interestingly, the differences in the concentrations of IL‐10 between the groups were insignificant (*p* = 0.065) (Figure 7L).

## 4 DISCUSSION

By employing network pharmacology and experimental validation, this work investigated the pharmacological effects of hyperforin on VCI. We established a compound–target–pathway interaction network and proposed possible processes that account for hyperforin's positive effects on VCI. Then, a BCCAO mouse model was established to assess the impact of hyperforin on pathological changes in the brain and cognitive abilities in VCI mice. We further studied the VEGFR_2_/SRC signaling pathway in vitro and found that hyperforin could attenuate the inflammatory activation of microglia after OGD/R treatment. Taken together, this study represents the first application of an integrative pharmacological approach to reveal that hyperforin may reduce the microglia‐induced inflammatory response and attenuate VCI through the VEGFR_2_/SRC signaling pathway.

*Hypericum perforatum* L. is extensively recognized for its efficacy in treating depression.^50^ Its applications have been expanding in recent years. Due to its complex composition, pinpointing the specific active ingredients of TCM poses a considerable challenge. Network pharmacology provides a valuable approach for screening and identifying the active components in TCM. By analyzing the correlation between diseases, targets, and components, the pharmacological mechanisms of the drugs can be further elucidated.^51^, ^52^ Our study revealed that hyperforin, the principal bioactive constituent of *H. perforatum* L., plays a crucial role in regulating VCI through the VEGFR_2_/SRC signaling pathway. Research has indicated that hyperforin exhibits a multitude of pharmacological properties, such as benefits against diabetes, depression, inflammation, dementia, tumors, and immunological regulation.^53^, ^54^ In AD models, hyperforin treatment has demonstrated the ability to partially protect against Aβ‐induced neurotoxicity, reduce astroglial and microglial reactions, and exhibit antioxidant and anti‐inflammatory properties.^23^ These findings suggest that hyperforin could have important implications in AD and other cognitive impairment‐related pathologies.^22^, ^23^

According to network pharmacology analysis, a BCCAO‐induced VCI model^55^ was used to explore the effects of hyperforin in vivo and discovered that intraventricular injection of hyperforin significantly improved cognitive impairment and alleviated depression‐like behaviors in VCI mice. Our results are in line with a prior meta‐analysis that *H. perforatum* L. could enhance cognition in rodents.^56^ Previous studies have demonstrated that hyperforin could improve poststroke depression and anxiety.^57^ White matter lesions are characteristic pathological changes of VCI and are considered important contributors to cognitive dysfunction in VCI mice.^34^ Consistent with our previous findings, we observed significant WMLs in the hippocampus and corpus callosum of VCI mice.^7^ However, after treatment with hyperforin, both WMLs and cognitive performance significantly improved. Considering our previous report on hyperforin promoting the maturation of oligodendrocytes,^49^ which are key targets in white matter, we believe that hyperforin has the potential to alleviate WMLs.

Recently, a model suggests that chronic stress and inflammation contribute to microglial activation, resulting in white matter damage and vascular and brain pathology.^58^ These impairments initially present as depression and mild cognitive impairment but may eventually progress to dementia. Our studies support the idea that microglial‐induced neuroinflammation can lead to WMLs and cognitive impairment. Microglia play a critical role in WMLs and an elevated inflammatory response parallel with WMLs,^59^, ^60^ as is shown in our study. Meanwhile, recent studies indicated that pro‐inflammatory activated microglia could exacerbate WMLs^61^ and release pro‐inflammatory markers.^62^, ^63^ Our research identified a noticeable activation of the hippocampal microglia and enhanced secretion of pro‐inflammatory cytokines in VCI mice, which was reversed by hyperforin treatment. Collectively, our findings demonstrated that treatment with hyperforin alleviated microglial activation, neuroinflammation, and WMLs, which may contribute to the cognition‐improving effect.

Then, how does hyperforin exert its anti‐inflammatory role? To address this question, we exposed BV2 microglia cells to OGD/R, which mimics microglial activation induced by ischemia in vitro.^64^ As the primary mediator, VEGFR_2_ takes on a pivotal function in the physiological and pathological effects of VEGFA. VEGFA can bind to VEGFR_2_ and subsequently control survival and vascular permeability through two distinct signaling pathways mediated by SRC activation.^14^ Additionally, studies have demonstrated that M1‐type microglia can secrete VEGFA, TNF‐α, and IL‐6 to promote angiogenesis via the HIF‐1α/VEGF/VEGFR_2_ pathway,^65^ and the overexpression of VEGFA induced by excessive hypoxia leads to excessive brain vascular permeability.^66^ Despite its beneficial effects such as facilitating reparative angiogenesis, and providing neuroprotection in the aftermath of ischemic stroke, VEGFA demonstrates certain limitations.^67^ VEGFA can also contribute to CNS pathology through multiple mechanisms including angiogenesis induction, microglia,^68^ and T cell^69^ stimulation. M1 microglia are recognized as contributors to inflammatory damage in ischemic stroke,^70^ AD,^71^ Parkinson's disease, and other nervous system diseases.^72^ In our study, both treatment with SU5416, a VEGFR_2_ inhibitor, and hyperforin noticeably inhibited the M1 polarization of OGD/R‐induced BV2 cells and suppressed the VEGFR_2_/SRC pathway. This suggests that hyperforin exerts its anti‐inflammatory properties by inhibiting the VEGFR_2_/SRC pathway. Moreover, treatment with SU5416 and hyperforin simultaneously downregulated p‐SRC/SRC and VEGFA expressions in OGD/R‐induced BV2 cells. Using SRC inhibitors in a model of ischemic brain damage has demonstrated significant reductions in SRC and VEGF expression, cerebral edema, infarct size, and neurological dysfunction.^73^ Subsequent research revealed that inhibiting SRC has a protective effect on CNS inflammation by reducing microglia‐induced neuroinflammation^74^ and phagocytosis.^75^

In summary, our findings support the conclusion that OGD/R‐induced BV2 cells secrete VEGFA, which subsequently binds to VEGFR_2_ receptors on the cell surface. This interaction activates the VEGFR_2_/SRC pathway and ultimately leads to the promotion of microglia M1‐polarization. This, in turn, leads to excessive release of VEGFA and pro‐inflammatory cytokines. Conversely, hyperforin can inhibit the VEGFR_2_/SRC pathway and suppress the cascade release of VEGFA and inflammatory factors. Ultimately, this may preserve the integrity of the white matter and the cognitive ability can be preserved in VCI mice. Moving forward, our future studies will delve deeper into investigating the role of SRC activation in VEGFA secretion within microglia.

## 5 CONCLUSION

In conclusion, the study suggests that hyperforin possesses the potential to modulate M1 polarization of microglia by inhibiting the VEGFR_2_/SRC signaling pathways, thereby alleviating neuroinflammation and WMLs induced by BCCAO in a mouse model. This, in turn, may lead to cognitive improvement in VCI mice. However, it is important to note that our present study has several limitations. To gain a comprehensive understanding, further investigations are warranted to explore the downstream pathways associated with SRC activation and investigate the underlying mechanisms by which M1 microglia contribute to WMLs in vivo and in vitro.

## AUTHOR CONTRIBUTIONS

Xin Gao: Methodology, performed the experiments, and Writing—original draft. Jingjing Chen: Methodology, performed the experiments, and Writing—original draft. Ge Yin, Yanqun Liu, Riu Sun: Methodology, performed the experiments. Zhengsheng Gu, Xuehao Jiao: Performed statistical analyses. Xu Sun, Ling Wang, Yuting Kan: Drew the figures. Nuo Wang: Collected data. Yanbo Zhang: Conceptualization. Xiaoying Bi, Bingying Du: Supervision, Conceptualization, Writing—review and editing.

## CONFLICT OF INTEREST STATEMENT

The authors declare no conflicts of interest.

## Supporting information


Data S1

## Data Availability

All data sets generated for this study are included in the article/Supplementary Material.
