# Supplementary material for: Hyperforin ameliorates neuroinflammation and white matter lesions by regulating microglial VEGFR2 /SRC pathway in vascular cognitive impairment mice
Source: CNS Neurosci Ther. 2024 Mar 11;30(3):e14666. doi: 10.1111/cns.14666 (PMC10927933; doi:10.1111/cns.14666)
Supplement: Supplementary file 1 — Data S1 [file CNS-30-e14666-s001.zip › Supplementary File.docx]

***Supplementary File***

**Hyperforin ameliorates neuroinflammation and white matter lesions by regulating microglial VEGFR_2_/SRC pathway in vascular cognitive impairment mice**

Xin Gao^1^, Jingjing Chen^1^, Ge Yin^1^, Yanqun Liu^1^, Zhengsheng Gu^1^, Rui Sun^1^, Xu Sun^1^, Xuehao Jiao^1^, Ling Wang^1^, Nuo Wang^1^, Yanbo Zhang^2^, Yuting Kan^1^, Xiaoying Bi^1,^*, Bingying Du^1,^*

^1^ Department of Neurology, Shanghai Changhai Hospital, Second Military Medical University/Naval Medical University, Shanghai, 200433, China.

^2^ Department of Psychiatry, Faculty of Medicine and Dentistry, University of Alberta, Edmonton, AB, Canada

*Correspondence authors:

Bingying Du*, E-mail: 15800614142@163.com

Xiaoying Bi*, E-mail: bixiaoying2013@163.com

The first two authors contributed equally to this work

Authors’ email addresses

Xin Gao^1^, E-mail: gaoxin_97@163.com

Jingjing Chen^1^, E-mail: chenjingjing950103@163.com

Ge Yin, E-mail: yg9561@163.com

Yanqun Liu, E-mail: liuyanqun588@sina.com

Zhengsheng Gu, E-mail: gzs1996@smmu.edu.cn

Rui Sun, E-mail: ricosun_hp@163.com

Xu Sun，E-mail: sunxu158@163.com

Xuehao Jiao, E-mail: jiaoxuehao@smmu.edu.cn

Ling Wang, E-mail: Lingwang1101@163.com

Nuo Wang, E-mail: 724128156@qq.com

Yanbo Zhang, E-mail: yanbo.zhang@ualberta.ca

Yuting Kan, E-mail: 1193257017@qq.com

Xiaoying Bi*, E-mail: bixiaoying2013@163.com

Bingying Du*, E-mail: 15800614142@163.com

**Supplement Table 1** The active components of the *Hypericum perforatum* discovered by TCMSP and TCIMD.

| Pubchem ID | Mol ID from TCMSP | Components | SMILE | MW | AlogP | DL | Hdon | Hacc |
| --- | --- | --- | --- | --- | --- | --- | --- | --- |
| 5281672 | MOL002008 | myricetin | C1=C(C=C(C(=C1O)O)O)C2=C(C(=O)C3=C(C=C(C=C3O2)O)O)O | 318.25 | 1.24 | 0.31 | 6 | 8 |
| 5281600 | MOL002037 | Amentoflavone | C1=CC(=CC=C1C2=CC(=O)C3=C(O2)C(=C(C=C3O)O)C4=C(C=CC(=C4)C5=CC(=O)C6=C(C=C(C=C6O5)O)O)O)O | 538.48 | 4.36 | 0.65 | 6 | 10 |
| [1794427](http://pubchem.ncbi.nlm.nih.gov/summary/summary.cgi?cid=1794427) | MOL001955 | Heriguard | C1[C@H]([C@H]([C@@H](C[C@@]1(C(=O)O)O)OC(=O)/C=C/C2=CC(=C(C=C2)O)O)O)O | 354.34 | -0.42 | 0.33 | 6 | 9 |
| 448437 | MOL002742 | LUT | CC1=C(C(C[C@H](C1)O)(C)C)/C=C/C(=C/C=C/C(=C/C=C/C=C(\C)/C=C/C=C(\C)/C=C/[C@H]2C(=C[C@@H](CC2(C)C)O)C)/C)/C | 568.96 | 9.47 | 0.54 | 2 | 2 |
| 12303645 | MOL000359 | sitosterol | CC[C@H](CC[C@@H](C)[C@H]1CC[C@@H]2[C@@]1(CC[C@H]3[C@H]2CC=C4[C@@]3(CC[C@H](C4)O)C)C)C(C)C | 414.79 | 8.08 | 0.75 | 1 | 1 |
| 5280805 | MOL000415 | rutin | C[C@H]1[C@@H]([C@H]([C@H]([C@@H](O1)OC[C@@H]2[C@H]([C@@H]([C@H]([C@@H](O2)OC3=C(OC4=CC(=CC(=C4C3=O)O)O)C5=CC(=C(C=C5)O)O)O)O)O)O)O)O | 610.57 | -1.45 | 0.68 | 10 | 16 |
| 5280863 | MOL000422 | kaempferol | C1=CC(=CC=C1C2=C(C(=O)C3=C(C=C(C=C3O2)O)O)O)O | 286.25 | 1.77 | 0.24 | 4 | 6 |
| 5280445 | MOL005930 | luteolin | C1=CC(=C(C=C1C2=CC(=O)C3=C(C=C(C=C3O2)O)O)O)O | 286.25 | 2.07 | 0.73 | 4 | 6 |
| 5280459 | MOL000701 | quercitrin | C[C@H]1[C@@H]([C@H]([C@H]([C@@H](O1)OC2=C(OC3=CC(=CC(=C3C2=O)O)O)C4=CC(=C(C=C4)O)O)O)O)O | 448.41 | 0.3 | 0.74 | 7 | 11 |
| [5280343](http://pubchem.ncbi.nlm.nih.gov/summary/summary.cgi?cid=5280343) | MOL000098 | quercetin | C1=CC(=C(C=C1C2=C(C(=O)C3=C(C=C(C=C3O2)O)O)O)O)O | 302.25 | 1.5 | 0.28 | 5 | 7 |
| 441298 | N/A | hyperforin | CC(C)C(=O)[C@]12C(=O)C(=C([C@](C1=O)(C[C@@H]([C@@]2(C)CCC=C(C)C)CC=C(C)C)CC=C(C)C)O)CC=C(C)C | 536.39 | 9.6 | N/A | 1 | 4 |
| 3663 | N/A | hypericin | CC1=CC(=O)C2=C(C3=C(C=C(C4=C3C5=C2C1=C6C(=CC(=O)C7=C(C8=C(C=C(C4=C8C5=C67)O)O)O)C)O)O)O | 504.08 | 5.7 | N/A | 6 | 8 |
| 5281643 | N/A | hyperin | C1=CC(=C(C=C1C2=C(C(=O)C3=C(C=C(C=C3O2)O)O)O[C@H]4[C@@H]([C@H]([C@H]([C@H](O4)CO)O)O)O)O)O | 464.1 | 0.4 | N/A | 8 | 12 |
| 5318391 | N/A | hypodematine | C1=CC=C(C=C1)/C/2=C/C=C\C=C/3\C=C(C=CC3=N2)O | 247.1 | 3.6 | N/A | 1 | 2 |
| 5284419 | N/A | Methylhesperidin | C[C@H]1[C@@H]([C@H]([C@H]([C@@H](O1)OC[C@@H]2[C@H]([C@@H]([C@H]([C@@H](O2)OC3=CC(=C4C(=O)C[C@H](OC4=C3)C5=CC(=C(C=C5)OC)OC)O)O)O)O)O)O)O | 624.21 | -0.8 | N/A | 7 | 15 |
| 10414856 | N/A | 3,8''-biapigenin | C1=CC(=CC=C1C2=CC(=O)C3=C(O2)C(=C(C=C3O)O)C4=C(OC5=CC(=CC(=C5C4=O)O)O)C6=CC=C(C=C6)O)O | 538.5 | 5 | N/A | 6 | 10 |
| 21590529 | N/A | Methyl (1S,3R,4R,5S)-4-methyl-1,5-bis(3-methylbut-2-enyl)-4-(4-methylpent-3-enyl)-3-(2-methylpropanoyl)-2-oxocyclohexane-1-carboxylate | CC(C)C(=O)[C@@H]1C(=O)[C@@](C[C@@H]([C@@]1(C)CCC=C(C)C)CC=C(C)C)(CC=C(C)C)C(=O)OC | 458.7 | 8.3 | N/A | 0 | 4 |
| 5281751 | N/A | pseudohypericin | CC1=CC(=C2C3=C4C5=C(C(=CC(=C5C2=O)O)O)C6=C7C4=C8C(=C13)C(=CC(=C8C(=O)C7=C(C=C6O)O)O)CO)O | 520.08 | 5.6 | N/A | 7 | 9 |


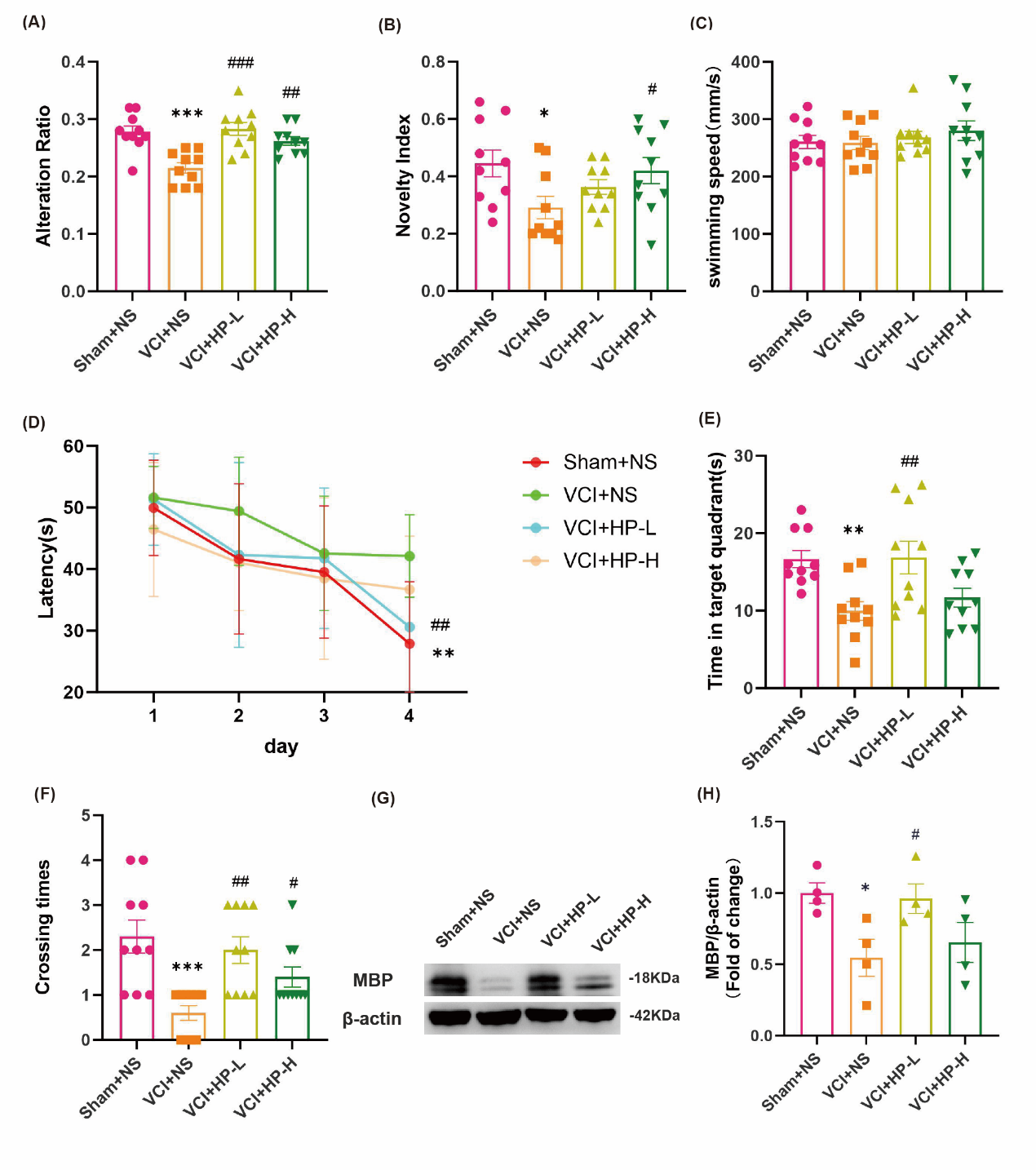


**Supplement Figure 1 The lower dose of hyperforin exhibited superior efficacy in ameliorating cognitive impairment induced by BCCAO** (A) The Alternations Ratio in the Y maze test. (B) The Novelty Index in the Y maze test. (C) The swimming speed of the mice. (D) The escape latency period during the training phase from day1 to day4. (E) The time spent in the target quadrant of MWM on day 5. (F) The crossings times of the platform during the MWM test on day 5. (G-H) Immunoreactive bands and quantitative analysis of Western blot of MBP and β-actin. Data are expressed as mean ± SEM (n = 5). * *P* < 0.05, ** *P* < 0.01 and *** *P* < 0.001, compared with Sham+NS group; ^＃^*P* < 0.05, and ^＃＃^*P* < 0.01 compared with VCI+NS group.


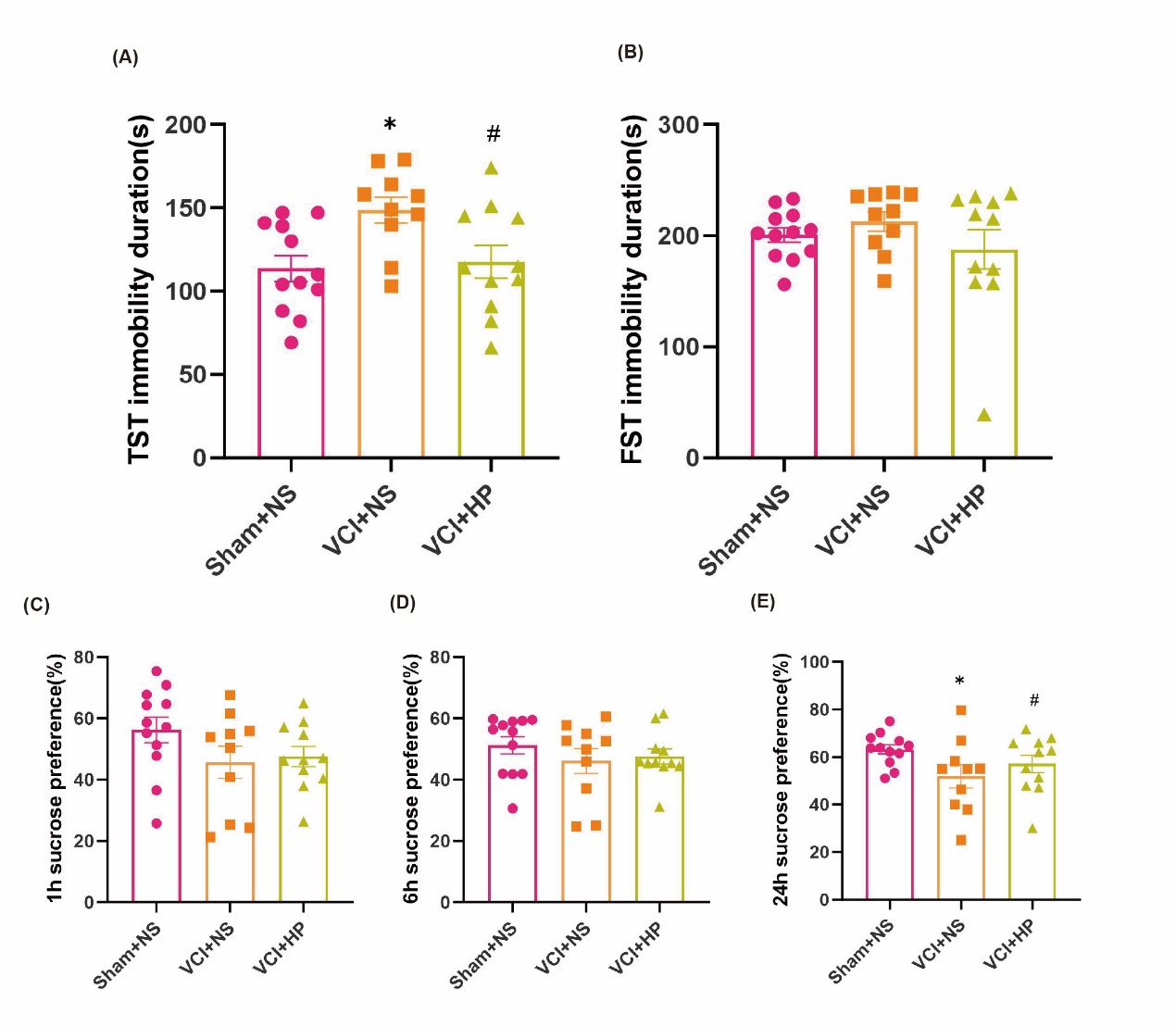


**Supplement Figure 2 Hyperforin alleviates the depressive behaviors in VCI mice.** (A) TST immobility durations. (B) FST immobility durations. (C- E). 1hour, 6hour, 24hour sucrose preference. Data are expressed as mean ± SEM (n = 10-12). * *P* < 0.05, compared with Sham+NS group; ^＃^*P* < 0.05, compared with VCI+NS group.


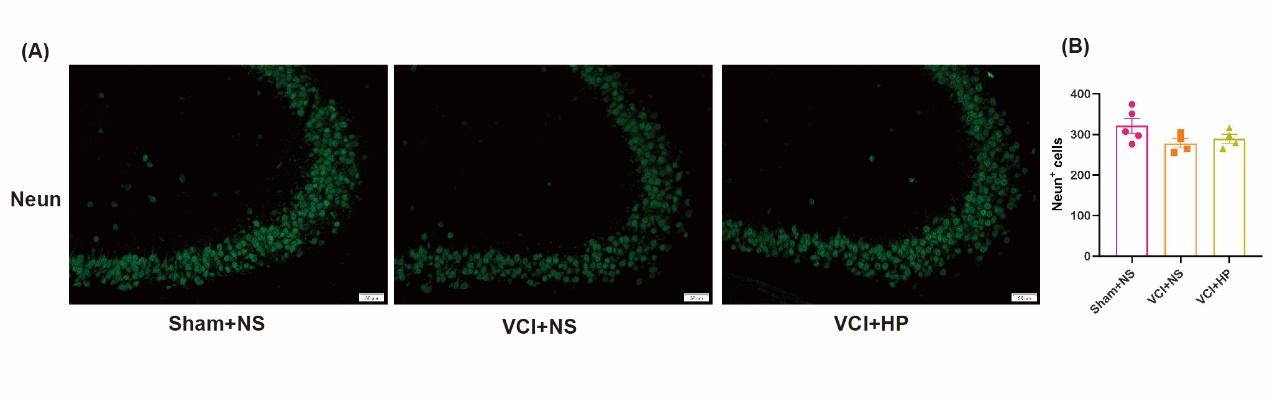


**Supplement Figure 3 The Neun immunostaining in hippocampal CA3 area.** (A) Representative images of Neun immunostaining in hippocampal CA3 area. (B) Quantification analysis of Neun+ cells in hippocampal CA3 area. Data are expressed as the mean ± SEM. (n = 5-6).


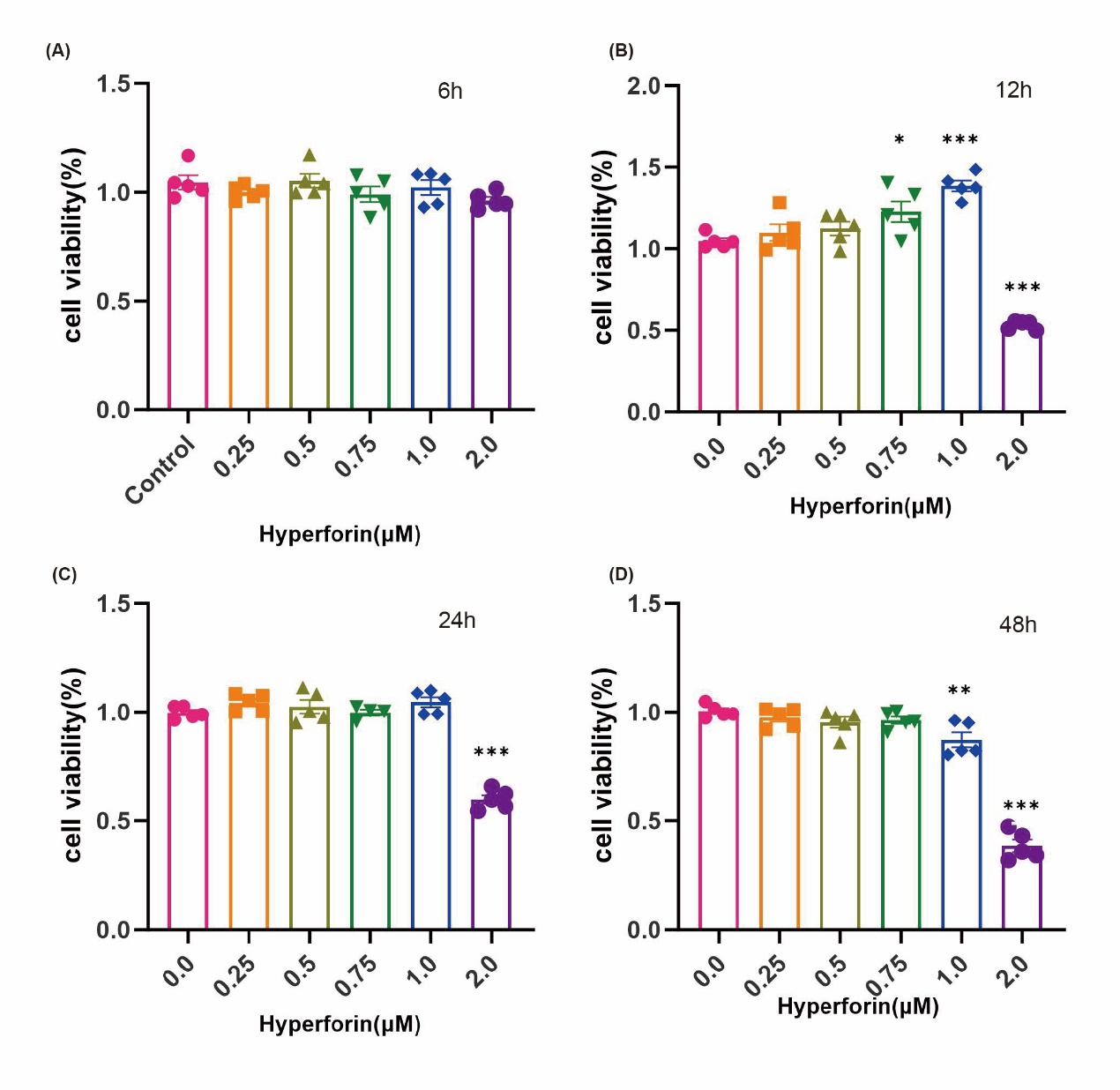


**Supplement Figure 4** **Cytotoxic or protective properties of hyperforin in the BV2 microglia cells.** (A-D) Changes in cell viability after 6-, 12-, 24, and 48-hour administration of hyperforin (0, 0.25, 0.5, 0.75, 1.0, and 2.0μM). The results are represented as the mean ± SEM (n = 6). * *P* < 0.05, ** *P* < 0.01, *** *P* < 0.001 as compared with vehicle group.
