# Supplementary material for: Hyperforin ameliorates neuroinflammation and white matter lesions by regulating microglial VEGFR2 /SRC pathway in vascular cognitive impairment mice
Source: CNS Neurosci Ther. 2024 Mar 11;30(3):e14666. doi: 10.1111/cns.14666 (PMC10927933; doi:10.1111/cns.14666)
Supplement: Supplementary file 1 — Data S1 [file CNS-30-e14666-s001.zip › the original image of the uncropped gelblot_1.pdf]

Full unedited gel/blot for Figure 4E

MBP

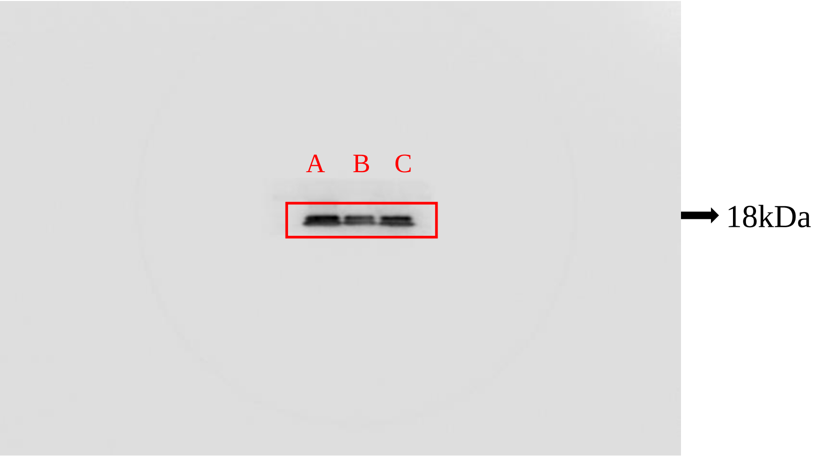

β-actin

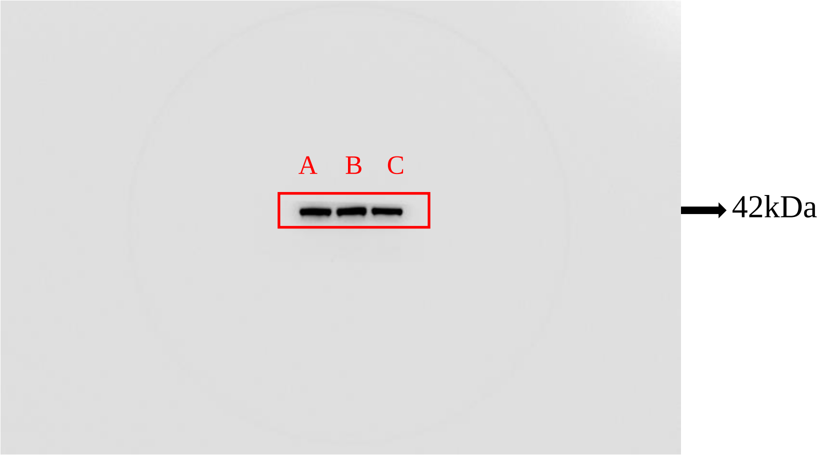

A=Sham+NS, B=VCI+NS, C=VCI+HP

Full unedited gel/blot for Figure 6A

VEGFR2

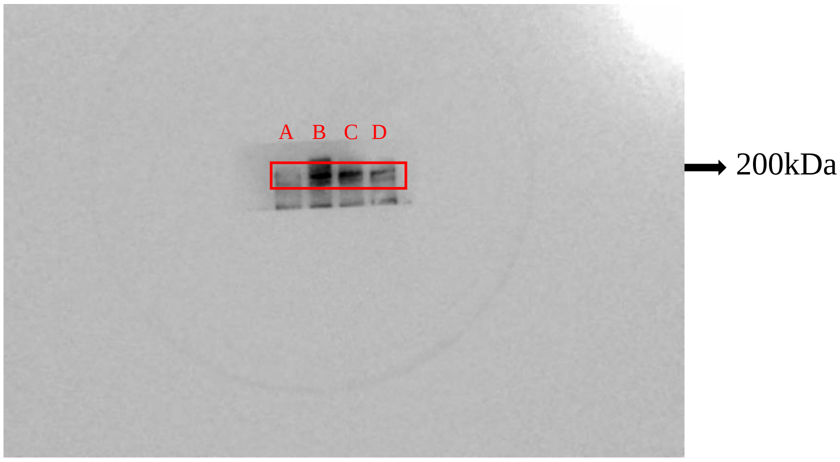

200kDa

VEGFA

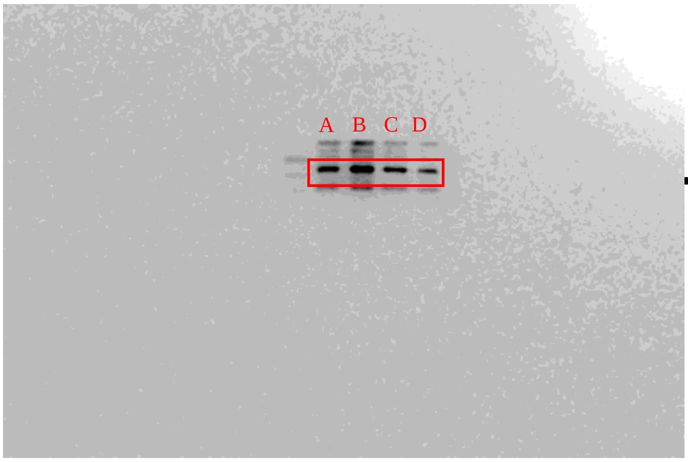

27kDa

β-actin

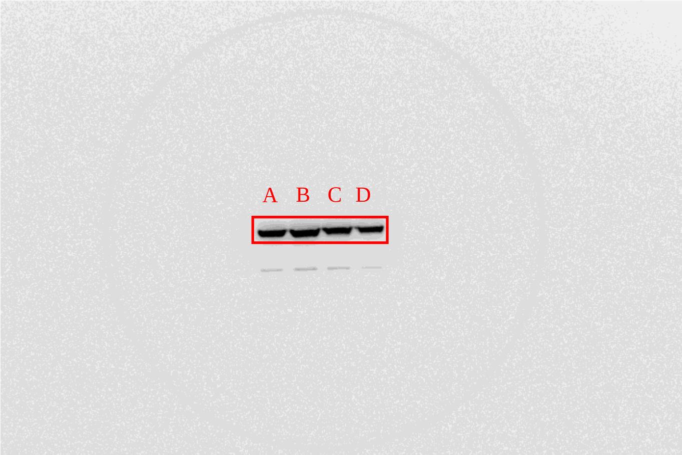

42kDa

A=Control, B=OGD/R, C=OGD/R+5μM SU5416, D= OGD/R+10μM SU5416

Full unedited gel/blot for Figure 6A

P-SRC

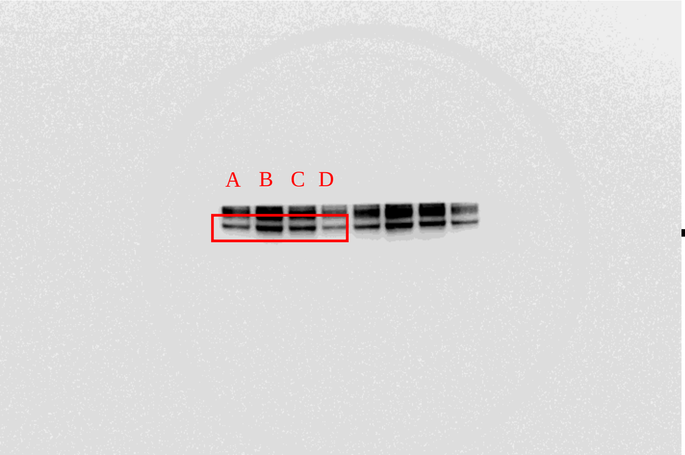

→ 60kDa

SRC

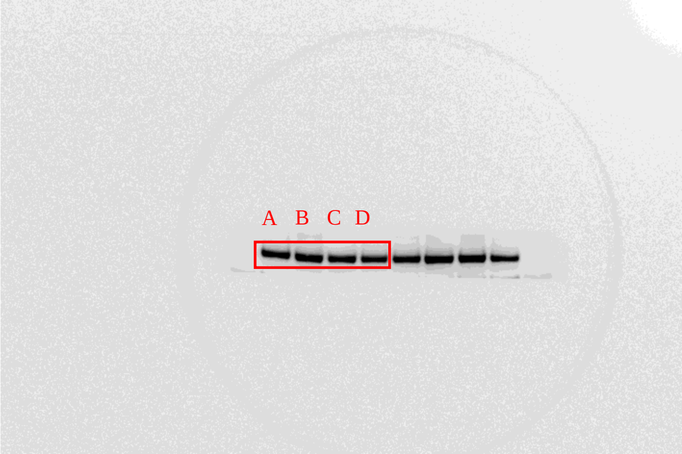

→ 60kDa

β-actin

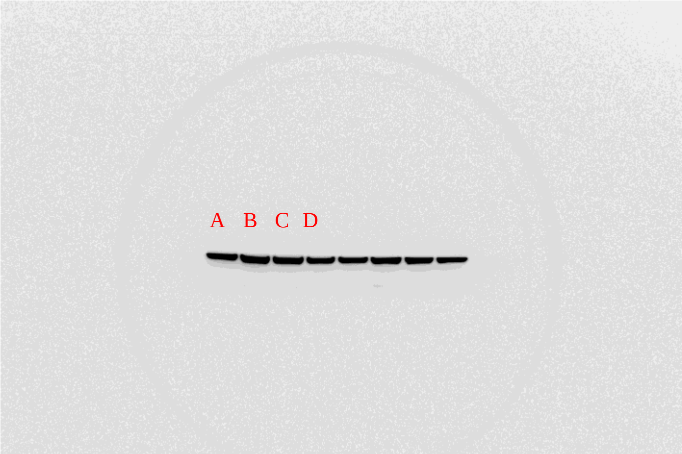

→ 42kDa

A=Control, B=OGD/R, C=OGD/R+5μM SU5416, D= OGD/R+10μM SU5416

Full unedited gel/blot for Figure 6E

i-NOS

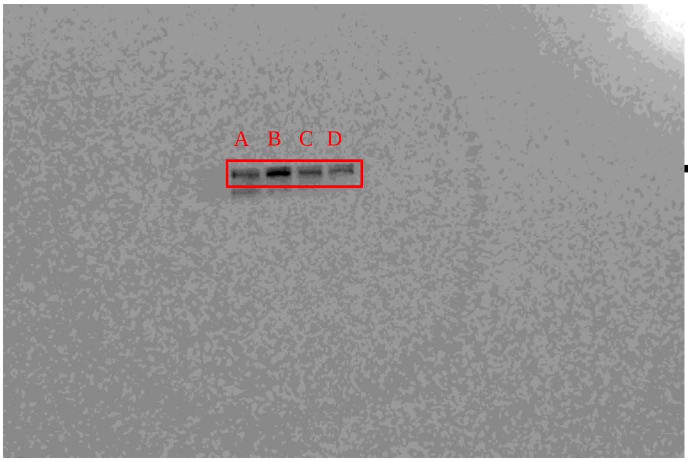

130kDa

CD86

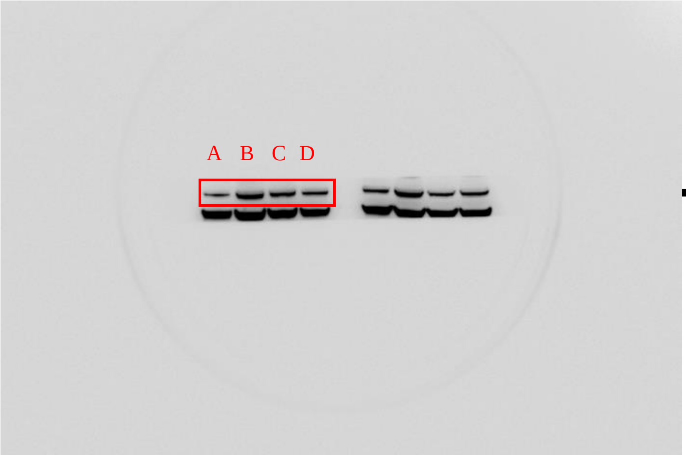

80kDa

ARG1

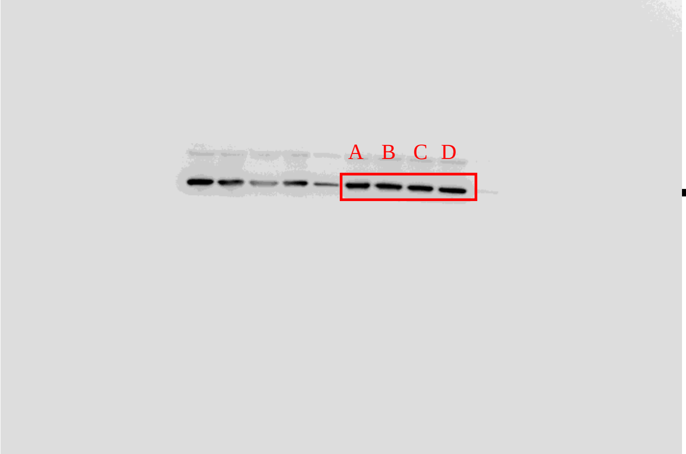

35kDa

β-actin

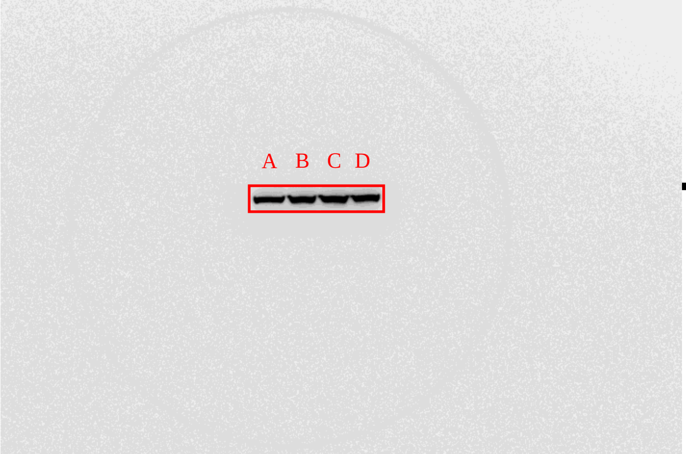

42kDa

A=Control, B=OGD/R, C=OGD/R+5μM SU5416, D= OGD/R+10μM SU5416

Full unedited gel/blot for Figure 6E

CD86

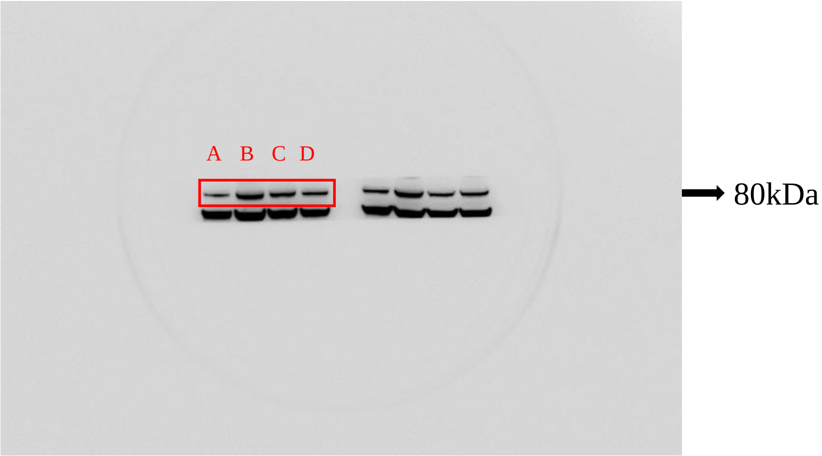

ARG1

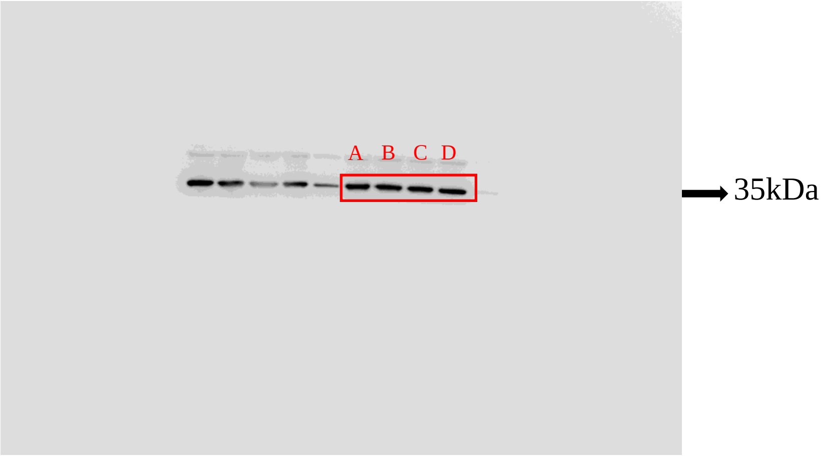

β-actin

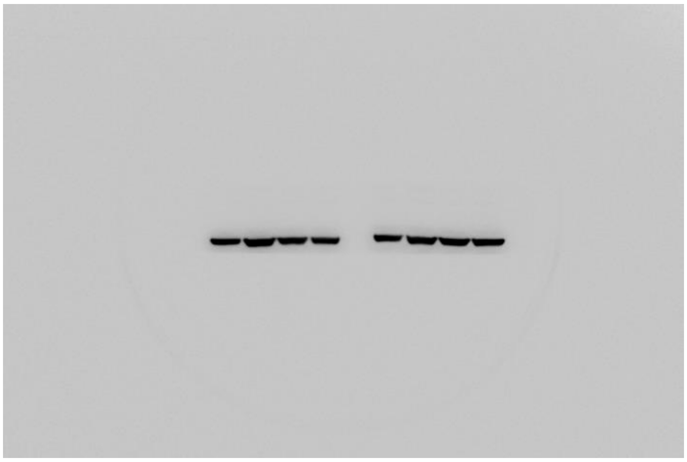

A=Control, B=OGD/R, C=OGD/R+5μM SU5416, D= OGD/R+10μM SU5416

Full unedited gel/blot for Figure 7A

VEGFR2

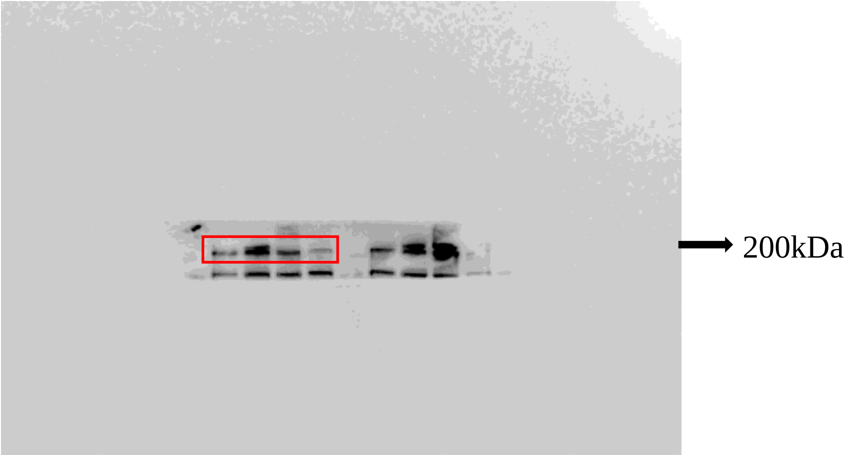

200kDa

VEGFA

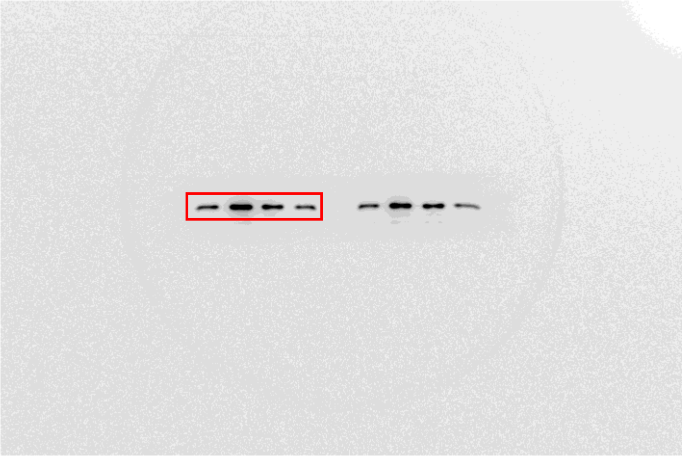

27kDa

β-actin

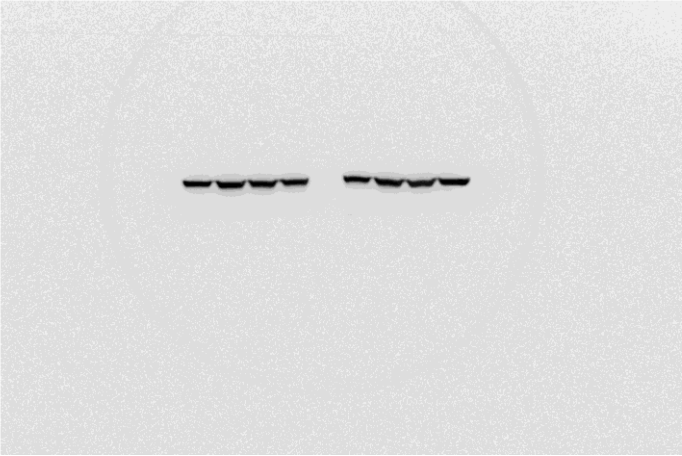

42kDa

A=Control, B=OGD/R, C=OGD/R+0.5μM HP, D= OGD/R+1.0μM HP

Full unedited gel/blot for Figure 7A

P-SRC

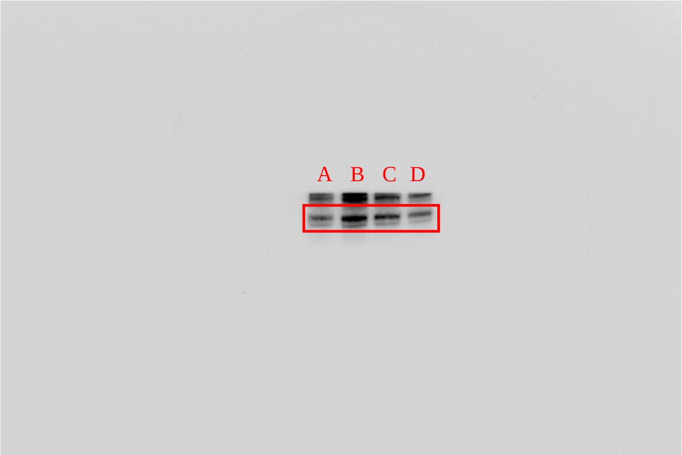

60kDa

SRC

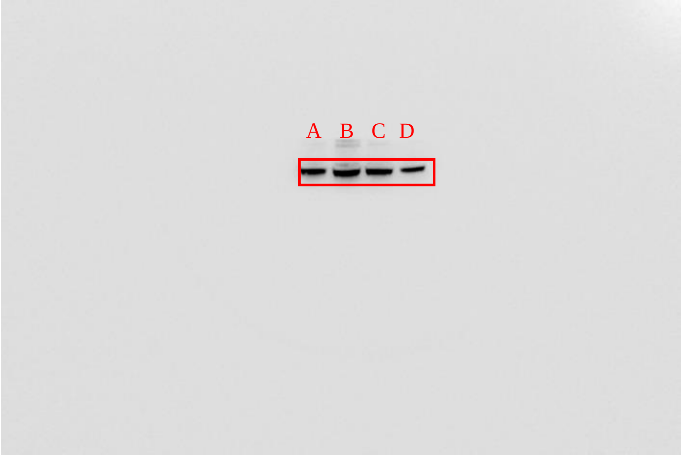

60kDa

β-actin

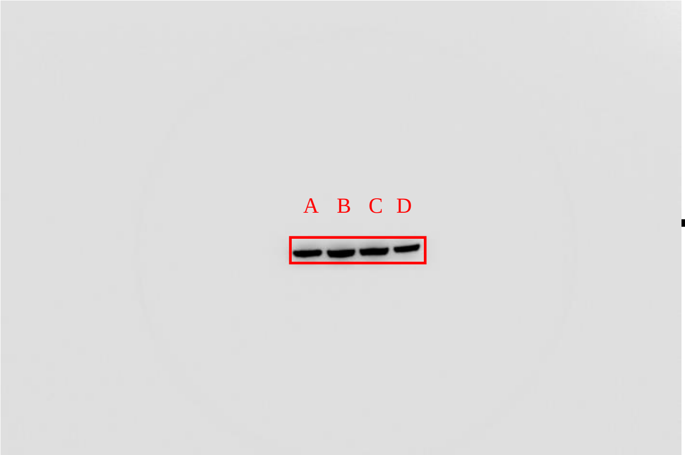

42kDa

A=Control, B=OGD/R, C=OGD/R+0.5μM HP, D= OGD/R+1.0μM HP

Full unedited gel/blot for Figure 7E

i-NOS

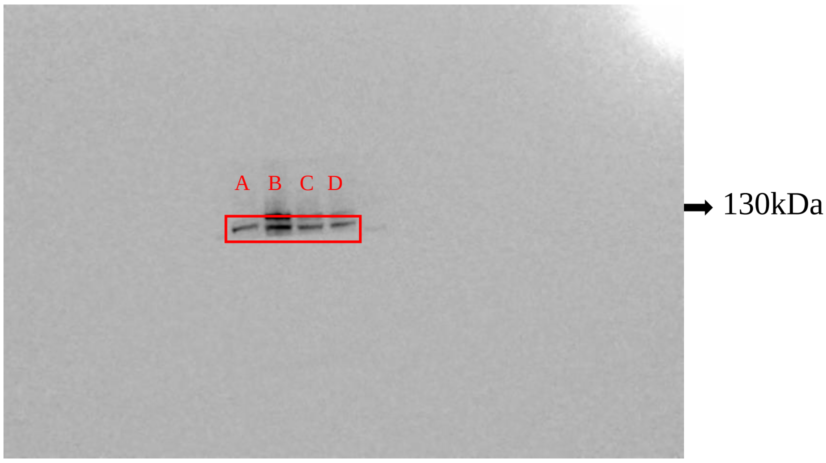

130kDa

CD86

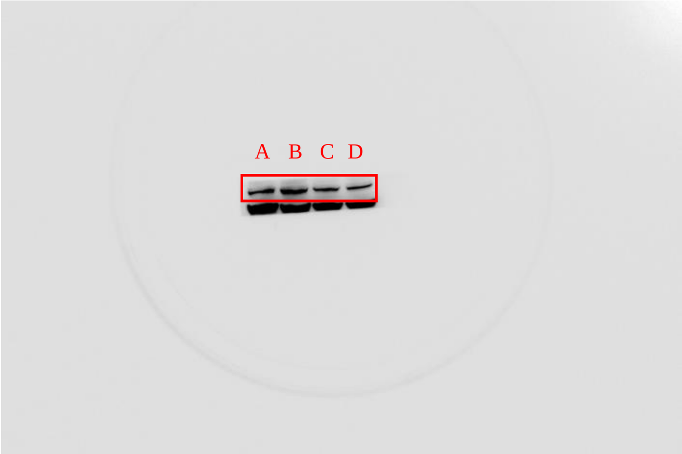

80kDa

ARG1

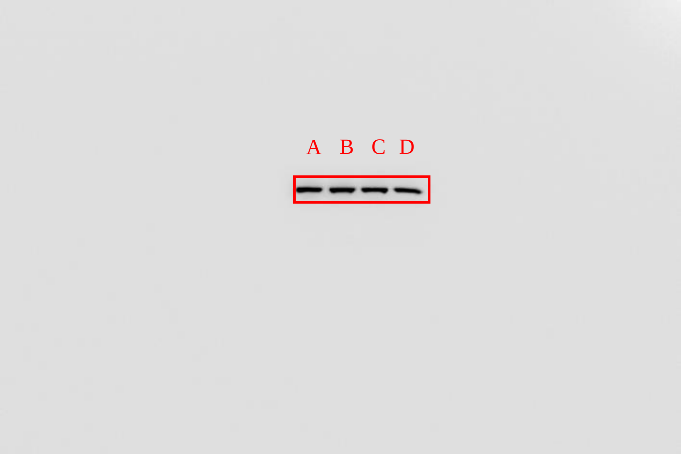

35kDa

β-actin

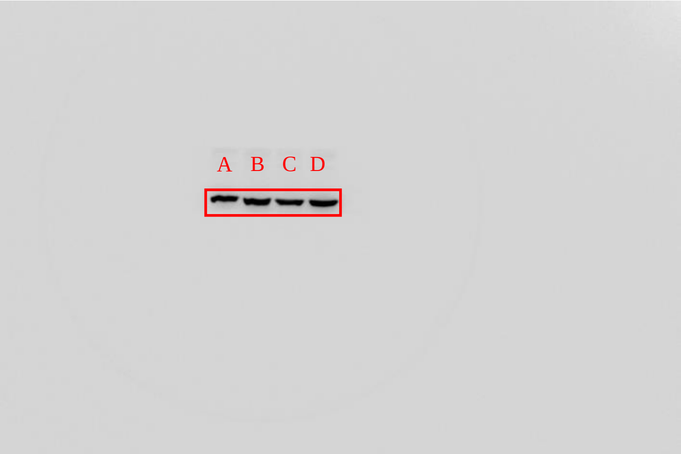

42kDa

A=Control, B=OGD/R, C=OGD/R+0.5μM HP, D= OGD/R+1.0μM HP

Full unedited gel/blot for Supplement Figure 1G

MBP

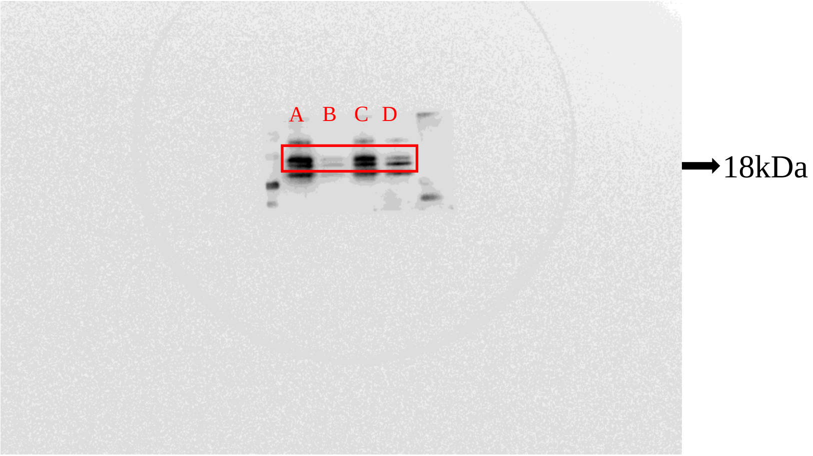

β-actin

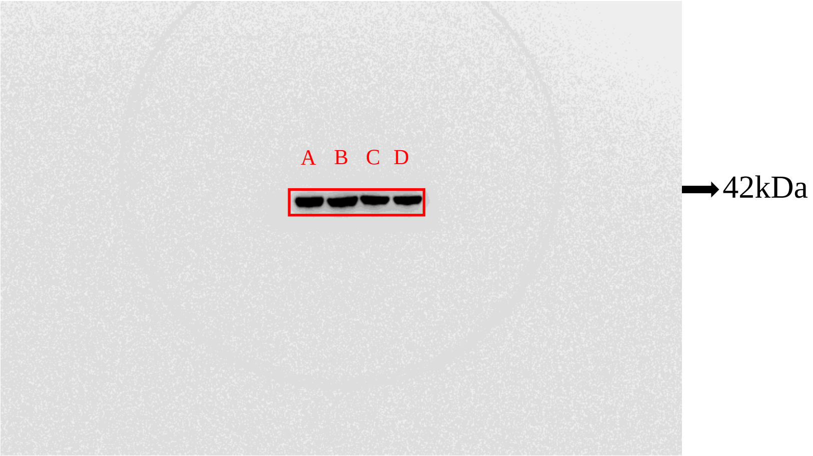

A=Sham+NS, B=VCI+NS, C=VCI+HP-L, D=VCI+HP-H
